# Angiotensin II participates in mitochondrial thermogenic functions via the activation of glycolysis in chemically induced human brown adipocytes

**DOI:** 10.1038/s41598-024-61774-0

**Published:** 2024-05-11

**Authors:** Yukimasa Takeda, Toshikazu Yoshikawa, Ping Dai

**Affiliations:** 1 Department of Cellular Regenerative Medicine, Graduate School of Medical Science, Kyoto Prefectural University of Medicine, 465 Kajii-cho, Kawaramachi-Hirokoji, Kamigyo-ku, Kyoto, 602-8566 Japan; 2 Louis Pasteur Center for Medical Research, 103-5 Tanaka-Monzen-cho, Sakyo-ku, Kyoto, 606-8225 Japan; 3 Department of Molecular Gastroenterology and Hepatology, Graduate School of Medical Science, Kyoto Prefectural University of Medicine, 465 Kajii-cho, Kawaramachi-Hirokoji, Kamigyo-ku, Kyoto, 602-8566 Japan

**Keywords:** Brown adipocytes, Angiotensin II, UCP1, Mitochondria, Adaptive thermogenesis, Glycolysis, Cell biology, Molecular biology

## Abstract

Brown adipocytes are potential therapeutic targets for the prevention of obesity-associated metabolic diseases because they consume circulating glucose and fatty acids for heat production. Angiotensin II (Ang II) peptide is involved in the pathogenesis of obesity- and cold-induced hypertension; however, the mechanism underlying the direct effects of Ang II on human brown adipocytes remains unclear. Our transcriptome analysis of chemical compound-induced brown adipocytes (ciBAs) showed that the Ang II type 1 receptor (AGTR1), but not AGTR2 and MAS1 receptors, was expressed. The Ang II/AGTR1 axis downregulated the expression of mitochondrial uncoupling protein 1 (*UCP1*). The simultaneous treatment with β-adrenergic receptor agonists and Ang II attenuated *UCP1* expression, triglyceride lipolysis, and cAMP levels, although cAMP response element-binding protein (CREB) phosphorylation was enhanced by Ang II mainly through the protein kinase C pathway. Despite reduced lipolysis, both coupled and uncoupled mitochondrial respiration was enhanced in Ang II-treated ciBAs. Instead, glycolysis and glucose uptake were robustly activated upon treatment with Ang II without a comprehensive transcriptional change in glucose metabolic genes. Elevated mitochondrial energy status induced by Ang II was likely associated with *UCP1* repression. Our findings suggest that the Ang II/AGTR1 axis participates in mitochondrial thermogenic functions via glycolysis.

## Introduction

Excess energy intake has increased the incidence of obesity and type 2 diabetes epidemics. Obesity is a serious risk factor for pathogenic metabolic alterations, such as impaired glucose tolerance, insulin resistance, and chronic inflammation^1^. Brown adipose tissue (BAT) is responsible for adaptive non-shivering thermogenesis in mammals^2^. Brown adipocytes specialise in heat generation by consuming blood glucose and free fatty acids for cold acclimation^3^. Cold circumstances activate brown adipocytes by the secretion of norepinephrine (NE), an endogenous β-adrenergic receptor (βAR) agonist, from the terminals of sympathetic nerves^3^. Heat production is conferred by mitochondrial uncoupling protein 1 (UCP1) located on the inner membrane^4^. UCP1 relieves the electron proton gradient for heat generation, which is uncoupled from adenosine triphosphate (ATP) synthesis. BAT contributes not only to adaptive thermogenesis but also to the improvement of symptoms of metabolic syndrome by fuelling circulating blood glucose, free fatty acids, branched-chain amino acids, and acylcarnitin^5,6^. BAT functions as a metabolic sink and helps improve blood glucose, triglycerides, and high-density lipoprotein levels, thereby reducing the risk of cardiometabolic diseases^7^.

The renin-angiotensin system (RAS) regulates blood pressure, fluid, and electrolyte homeostasis and is involved in many physiological events in diverse organs^8^. The adipose tissue is known to serve as the primary source of angiotensinogen (AGT)^9^. Obesity is correlated with increased plasma levels of AGT, renin, angiotensin-converting enzyme (ACE), and aldosterone^10^. Human adipose tissue expresses the RAS components required for the responsiveness to angiotensin II (Ang II)^11^. Locally derived or circulating Ang II negatively regulates adipocyte differentiation and functions^12,13^. The anti-adipogenic effects of Ang II are due to the activation of the extracellular signal-regulated kinase (ERK) in the mitogen-activated protein kinase (MAPK) signalling pathway through the type 1 receptor, AGTR1^14,15^. In contrast, AGTR1-knockout rats showed ameliorated diet-induced obesity, elevated adipose lipolysis, and fatty acid oxidation^16^. This was caused by the activation of the cAMP/protein kinase A (PKA) pathway, which overlaps with the thermogenesis pathway initiated by βAR receptors^17^. In obesity, the RAS hyperactivation of the classical arm mediated by Ang II/AGTR1 is exacerbated and associated with obesity-induced hypertension, insulin resistance, and inflammation^18,19^. In humans, plasma AGT and leptin levels, blood pressure, and insulin secretion in response to oral glucose load are positively correlated with body mass index, suggesting a close relationship between the RAS and obesity^20^.

Clinical epidemiological studies have shown that chronic or intermittent cold exposure is a risk factor for hypertension and cardiovascular diseases^21,22^. Cold temperatures activate the sympathetic nervous system (SNS), leading to cold-induced hypertension (CIH) via the RAS^23,24^. The elevation of blood pressure during CIH is beneficial for circulatory function and metabolic rate during non-shivering thermogenesis^25–27^. Cold-induced increases in systolic blood pressure are largely abolished by treatment with losartan, a potent AGTR1 inhibitor^28^. An increase in hypothalamic AGTR1 expression is involved in the development of CIH, whereas the genetic ablation of AGTR1 attenuates CIH^29^. Notably, cold exposure elevated blood Ang II and NE levels in rats and human subjects^30–32^, implying that Ang II may be involved in the regulation of cold-induced thermogenesis. Ang II treatment increased Ang II content in rat interscapular BAT and facilitated the presynaptic release of NE from cold-exposed rat BAT^33^. Several reports have indicated that Ang II facilitated the synthesis and uptake of NE in adipose tissue through AGTR1^34,35^, indicating an association between Ang II and thermogenesis. Gene expression analysis indicated that thermogenic and energy metabolic genes, including *UCP1*, increased in the adipose tissues of chronic cold-exposed mice, which might be involved in the development of CIH^36^. Thus, Ang II is closely associated with cold-induced thermogenesis; however, the molecular mechanism underlying the direct action of Ang II in brown adipocytes has not been fully elucidated.

To overcome the difficulty of leveraging a sufficient amount of pure human brown adipocytes, we developed a method for the chemical conversion of the primary cultures of human dermal fibroblasts (HDFs) into brown adipocytes^37^. Chemical compound-induced brown adipocytes (ciBAs) were converted using a cocktail (RoFB) consisting of rosiglitazone, forskolin, and bone morphogenetic protein 7 (BMP7) in serum-free conditions, as previously reported^38^. Our previous study indicated that ciBAs expressed UCP1 more robustly than adipocytes differentiated from mesenchymal stem cells (MSCs)^39^. In addition, our transcriptome analysis indicated that a specific set of the RAS components was expressed in human brown adipocyte models, including ciBAs^39,40^. Our results suggest that the Ang II/AGTR1 axis contributes to thermogenic functions by mediating cellular lipid and glucose metabolism in human brown adipocytes.

## Results

### Ang II treatment represses UCP1 expression in human brown adipocyte models

**Figure 1:**
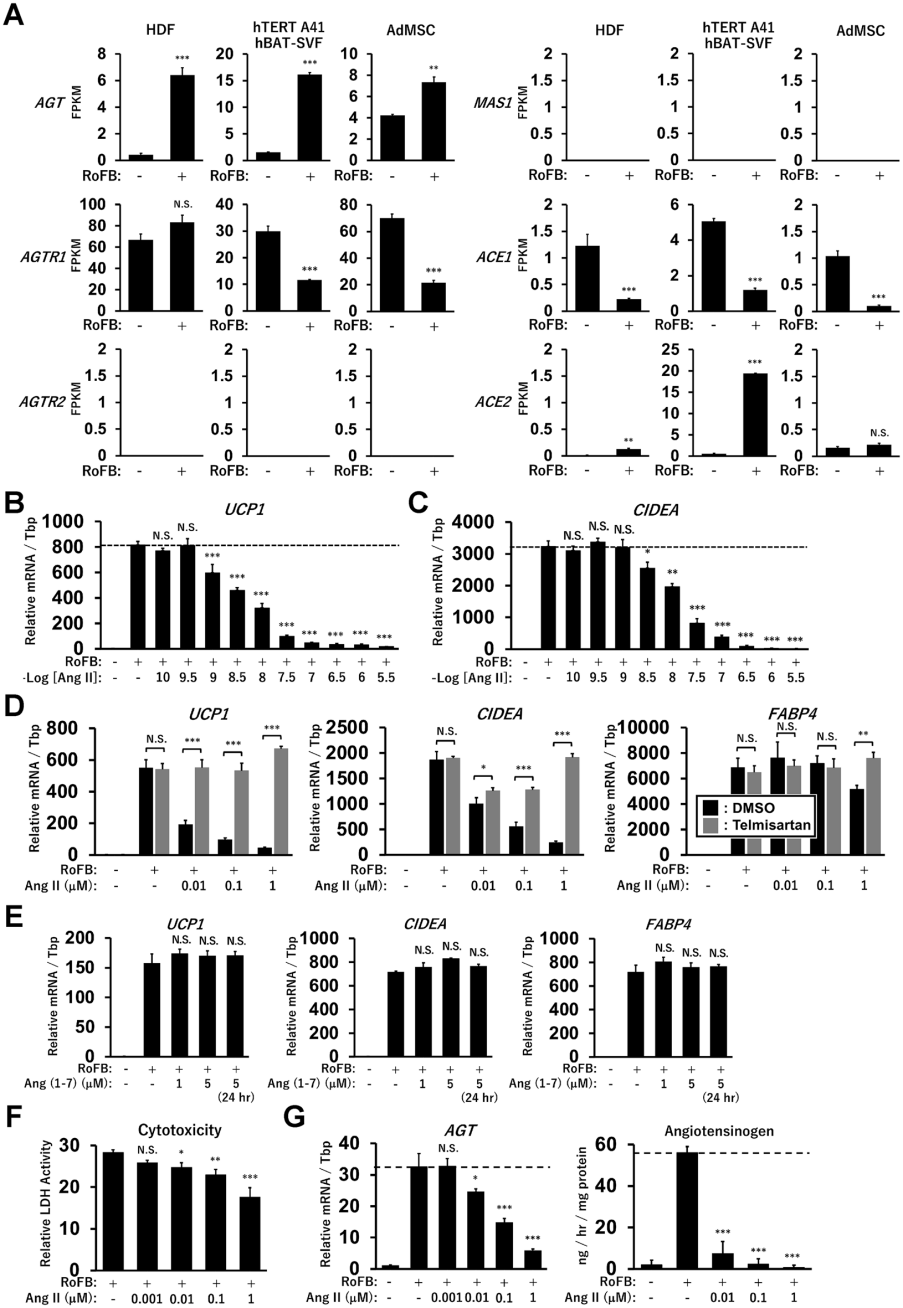
Repression of *UCP1* transcription by the Ang II/AGTR1 axis in human brown adipocyte models. (**A**) Our previous RNA-Seq analyses provide the mRNA level of the RAS components, including *AGT*, *AGTR1*, *AGTR2*, *MAS1*, *ACE1*, and *ACE2* between the controls and adipocytes converted from HDFs, hTERT A41hBAT-SVF, and AdMSCs. FPKM (fragments per kilobase of transcript per million mapped sequence reads) represents mRNA levels across genes and cell types. Statistical: two-tailed Student’s t-test. (**B,C**) The expression of *UCP1* and *CIDEA* mRNA was quantified by qRT-PCR in the ciBAs continuously treated with Ang II at concentrations of 0.1 nM to 3.16 μM, as indicated. (**D**) The expression of *UCP1*, *CIDEA*, and *FABP4* was quantified in ciBAs continuously treated with Ang II and telmisartan at 5 μM. Statistical analysis: two-tailed Student’s t-test. (**E**) The expression was quantified in ciBAs treated with Ang (1-7) peptide at indicated concentrations and incubation period (24 h). (**F**) The cytotoxicity of Ang II was assessed by measuring lactate dehydrogenase (LDH) activity in the culture supernatants. (**G**) AGT mRNA level and its secretion into the supernatant were quantified by qRT-PCR and ELISA, respectively, in ciBAs continuously treated with Ang II at indicated concentrations. Data represent mean ± SD (*n* = 3). *p*-values were determined using one-way ANOVA with Tukey’s multiple comparison test: * *p* < 0.05, ** *p* < 0.01, *** *p* < 0.001, N.S.; not significant.

Our previous transcriptome analysis showed that a specific set of the RAS components, including *AGT*, *AGTR1*, and *ACE1*, was expressed in human brown adipocyte models derived from HDFs, immortalised human preadipocytes (hTERT A41hBAT-SVF), and adipose tissue-derived MSCs (AdMSCs) (Fig. 1A). In contrast, *AGTR2* and the Mas receptor (*MAS1*) exhibited low expression levels, suggesting that the classical ACE1/Ang II/AGTR1 axis, but not the counter-regulatory ACE2/Ang (1-7)/MAS1 axis, could be a major RAS pathway in these adipocytes, including ciBAs. Although renin (*REN*) expression was limited to these adipocytes, the (pro)renin receptor, *ATP6AP2*, was abundantly expressed (Supplementary Fig. S1A). The low expression of chymase (*CAM1*) indicated that the ACE-independent synthesis of Ang II was negligible, whereas neprilysin (*NEP*) expression implied the possible synthesis of ANG (1–7) peptide from Ang I peptide. Next, to evaluate the effects of Ang II on thermogenic gene expression and brown adipogenesis, cells were continuously treated with Ang II during the chemical conversion of HDFs into ciBAs. Ang II strongly reduced the expression of *UCP1* and *CIDEA*, another brown adipocyte-enriched gene, in a dose-dependent manner at concentrations more than 1 nM (Fig. 1B,C). Incubation with Ang II for more than 3 days was required to elicit repressive effects on *UCP1* expression in ciBAs (Supplementary Fig. S1B–D). The repressive effects of Ang II were detected in ciBAs derived from other lines of HDFs and adipocytes derived from hTERT A41hBAT-SVF; however, relatively lower effects were detected in adipocytes derived from AdMSCs (Supplementary Fig. S2A–C). The repression of *UCP1*, *CIDEA*, and *FABP4* expression by Ang II was reversed by the addition of telmisartan, a potent inhibitor of AGTR1 (Fig. 1D). The expression of the adipocyte-enriched gene *FABP4* was slightly repressed at high concentrations of Ang II. The counter-regulatory peptide, Ang (1–7), an endogenous agonist of MAS1, had little effect on the expression, consistent with the low expression level of MAS1 (Fig. 1E). In addition, treatment with MLN-4760, a potent ACE2 inhibitor, had little effect on the repressive effects of Ang II (Supplementary Fig. S2D). Cytotoxicity was reduced in ciBAs treated with Ang II in a dose-dependent manner (Fig. 1F). Ang II treatment suppressed the expression and secretion of AGT in a dose-dependent manner (Fig. 1G). These results indicated that the Ang II treatment robustly repressed thermogenic *UCP1* transcription through the Ang II/AGTR1 axis in human brown adipocyte models.

### Ang II treatment reduces triglyceride lipolysis

**Figure 2:**
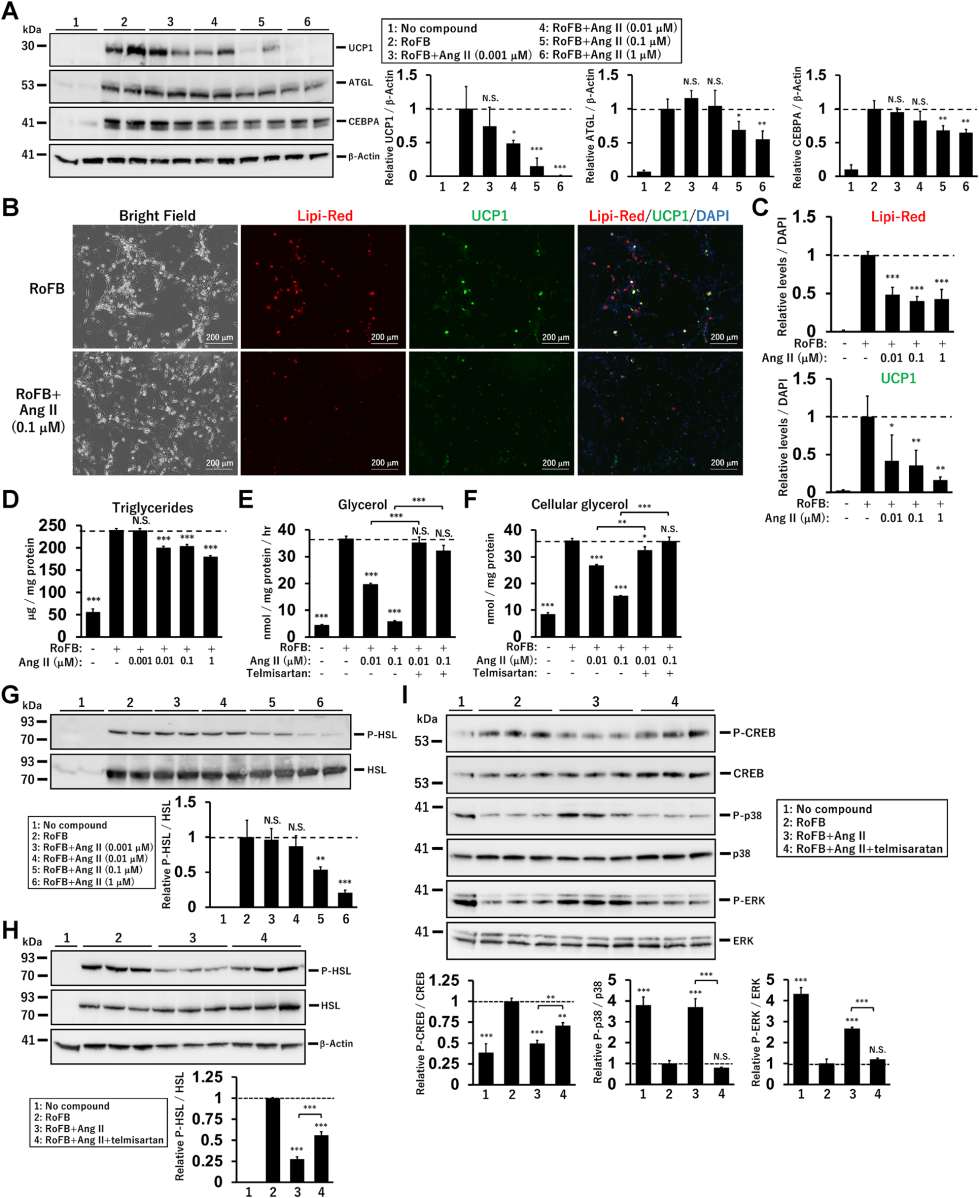
Repression of triglyceride lipolysis by Ang II in ciBAs. (**A**) UCP1, ATGL, CEBPA, and β-Actin proteins were immunoblotted in ciBAs continuously treated with Ang II at indicated concentrations. The relative protein levels normalised by β-Actin were calculated from corresponding band intensities. (**B**) Representative images of bright field, Lipi-Red staining (red), UCP1 protein (green), and merged image in the control ciBAs (RoFB) and ciBAs continuously treated with Ang II at 0.1 μM. The nuclei were visualised by DAPI (blue). Scale bars represent 200 μm. (**C**) The area of either Lipi-Red or UCP1 signals was quantified by ImageJ software and normalised by DAPI staining. (**D**) Cellular triglyceride content was measured in Ang II-treated ciBAs. (**E,F**) Glycerol secretion into the supernatant and cellular glycerol content were quantified in ciBAs continuously treated with Ang II and telmisartan. (**G**, **H**) The phosphorylated and total HSL proteins were quantified by immunoblotting in Ang II-treated ciBAs and ciBAs treated with Ang II (0.1 μM) and telmisartan (5 μM). The band intensities were measured by densitometry using ImageJ software. (**I**) The phosphorylation of CREB, P38, and ERK proteins was quantified by immunoblotting in ciBAs continuously treated with Ang II (0.1 μM) and telmisartan (5 μM). Data represent mean ± SD (*n* = 3–4). *p*-values were determined using one-way ANOVA with Tukey’s multiple comparison test: * *p* < 0.05, ** *p* < 0.01, *** *p* < 0.001, N.S.; not significant.

Lipid metabolism was characterised in Ang II-treated ciBAs. Similar to *UCP1* mRNA levels, protein expression was reduced by Ang II treatment in a dose-dependent manner (Fig. 2A). The protein levels of ATGL, a rate-limiting enzyme in adipocyte lipolysis, and CEBPA, a critical regulator of adipocyte differentiation, were repressed by Ang II treatment. Immunocytochemical analysis showed that fluorescent signals for lipid droplets and UCP1 protein were reduced by Ang II (Fig. 2B,C and Supplementary Fig. S3A). Consistently, cellular triglyceride storage was decreased by Ang II treatment (Fig. 2D). Notably, glycerol secretion in Ang II-treated ciBAs was robustly reduced, indicating that lipolysis was suppressed (Fig. 2E). The reduced glycerol secretion was reversed by simultaneous treatment with telmisartan. In addition, cellular glycerol levels were reduced by Ang II, implying that the reduction of glycerol secretion was unlikely to be due to increased glycerol uptake for recycling (Fig. 2F). The reduced phosphorylation of hormone-sensitive lipase (HSL) supported the reduced lipolysis in Ang II-treated ciBAs (Fig. 2G). The reduced phosphorylation of HSL was partially reversed by telmisartan treatment (Fig. 2H). Next, to address how Ang II regulates lipid metabolism, related signalling pathways were analysed. Ang II treatment reduced the phosphorylation of cAMP responsive element-binding protein (CREB), which was reversed by telmisartan treatment (Fig. 2I). In addition, Ang II enhanced the phosphorylation of the p38 and ERK MAPK signalling pathways. These results indicated that continuous treatment with Ang II reduced lipolysis through changes in several signalling pathways in ciBAs.

### Transient treatment with Ang II inhibits βAR-mediated UCP1 induction and lipolysis

**Figure 3:**
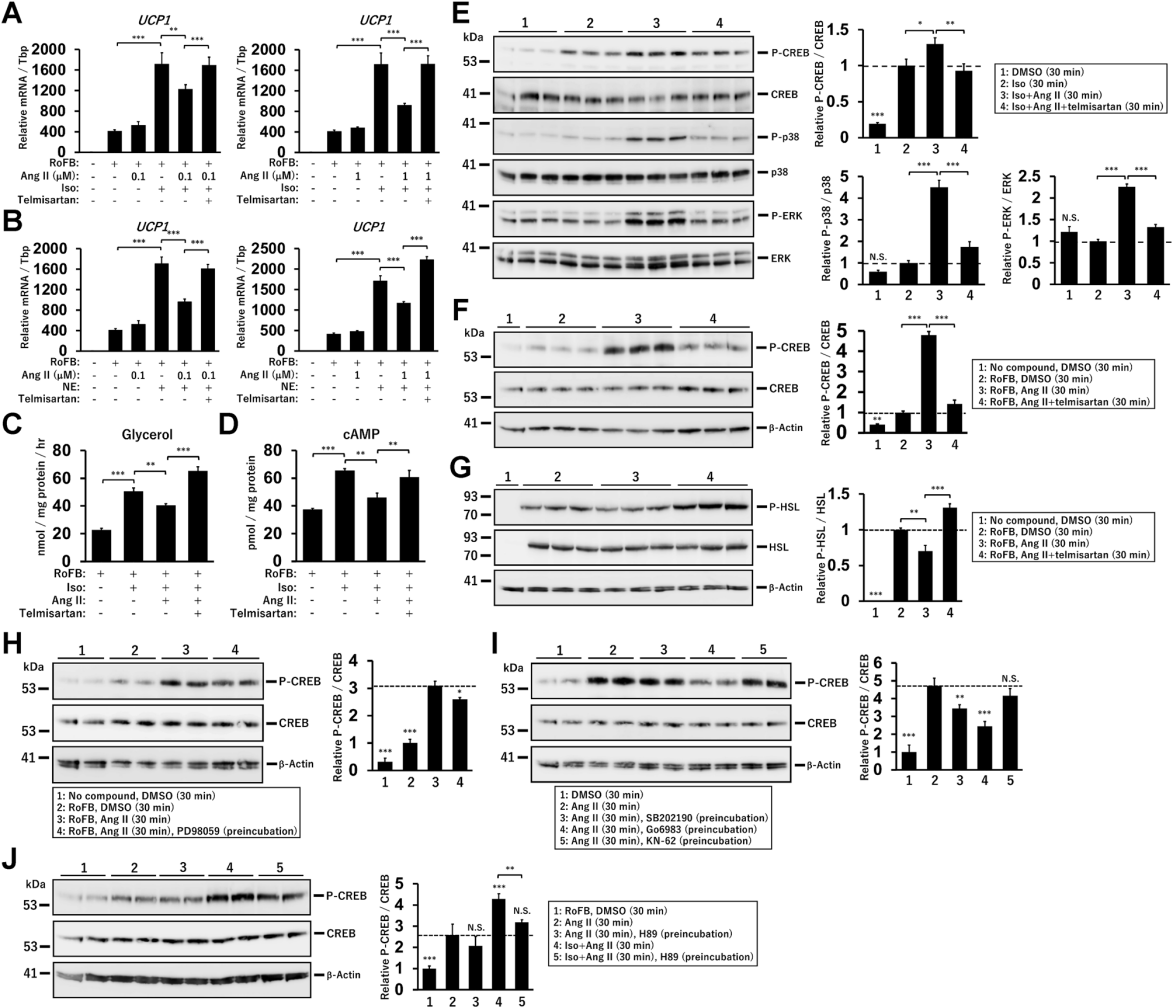
Inhibition of βAR-mediated UCP1 induction and lipolysis by Ang II in ciBAs. (**A,B**) The expression of UCP1 was measured 6 h after the treatment with either isopropanol (Iso) or norepinephrine (NE), Ang II (0.1 μM or 1.0 μM), and telmisartan as indicated. (**C**) Glycerol secretion was measured 60 min after the treatment with the combination of Iso, Ang II, and telmisartan. (**D**) Cellular cAMP levels were evaluated by ELISA 10 min after the treatment with the combination above. (**E**) The phosphorylation of CREB, P38, and ERK proteins was quantified by immunoblotting in ciBAs 30 min after the treatment with the combination as indicated. The band intensities were measured by densitometry using ImageJ software. (**F**, **G**) The phosphorylation of CREB and HSL proteins was quantified by immunoblotting 30 min after the treatment with Ang II and telmisartan as indicated. (**H**, **I**) The Ang II-induced phosphorylation of CREB was quantified 30 min after the treatment with Ang II in ciBAs preincubated with either PD98059, SB202190, Go6983, or KN-62 for 1 h. (**J**) The phosphorylation of CREB was quantified 30 min after the treatment with either Ang II or the combination with Iso and Ang II in ciBAs preincubated with H89 at 25 μM for 1 h. Data represent mean ± SD (*n* = 3–4). *p*-values were determined using one-way ANOVA with Tukey’s multiple comparison test: * *p* < 0.05, ** *p* < 0.01, *** *p* < 0.001, N.S.; not significant.

Under cold conditions, blood Ang II and NE levels increase via the activation of the SNS^30–32^. UCP1 expression and lipid lipolysis are activated for thermogenesis through the cAMP signalling pathway initiated by the activation of βAR by NE^41^. To evaluate the effects of Ang II on βAR-mediated thermogenesis, Ang II and either isoproterenol (Iso) or NE were simultaneously administered to ciBAs (Fig. 3A,B). The induced *UCP1* expression was partially inhibited by treatment with Ang II, and additional treatment with telmisartan reversed this inhibition. Iso-induced glycerol secretion and cellular cAMP levels were also reduced by Ang II treatment, which was reversed by the additional treatment with telmisartan (Fig. 3C,D). In contrast, transient treatment with Ang II further enhanced Iso-induced CREB phosphorylation (Fig. 3E). Transient treatment with Ang II increased p38 and ERK phosphorylation. Although continuous treatment reduced CREB phosphorylation, transient treatment was confirmed to robustly enhance the phosphorylation (Fig. 3F). In contrast, HSL phosphorylation was reduced by transient treatment (Fig. 3G). To identify upstream signalling pathways responsible for CREB phosphorylation by Ang II, several kinase inhibitors were pre-incubated before Ang II treatment. PD98059, an inhibitor of the ERK MAPK pathway, caused a slight reduction in the phosphorylation (Fig. 3H). Pre-incubation with SB202190, an inhibitor of the p38 MAPK pathway, also slightly decreased this effect, whereas Go6983, a potent inhibitor of pan-protein kinase C (PKC), further reduced it (Fig. 3I). KN-62, an inhibitor of calmodulin-dependent protein kinase II (CaMKII), had little effect on the phosphorylation. In addition, H89, a potent inhibitor of PKA, did not strongly affect the Ang II-mediated CREB phosphorylation, whereas H89 repressed Iso-mediated CREB phosphorylation (Fig. 3J). These results indicated that Ang II treatment inhibited βAR-mediated UCP1 expression and lipolysis by partially interfering with the thermogenesis pathway through AGTR1.

### Ang II treatment enhances mitochondrial oxygen consumption rate (OCR) through the activation of glycolysis

**Figure 4:**
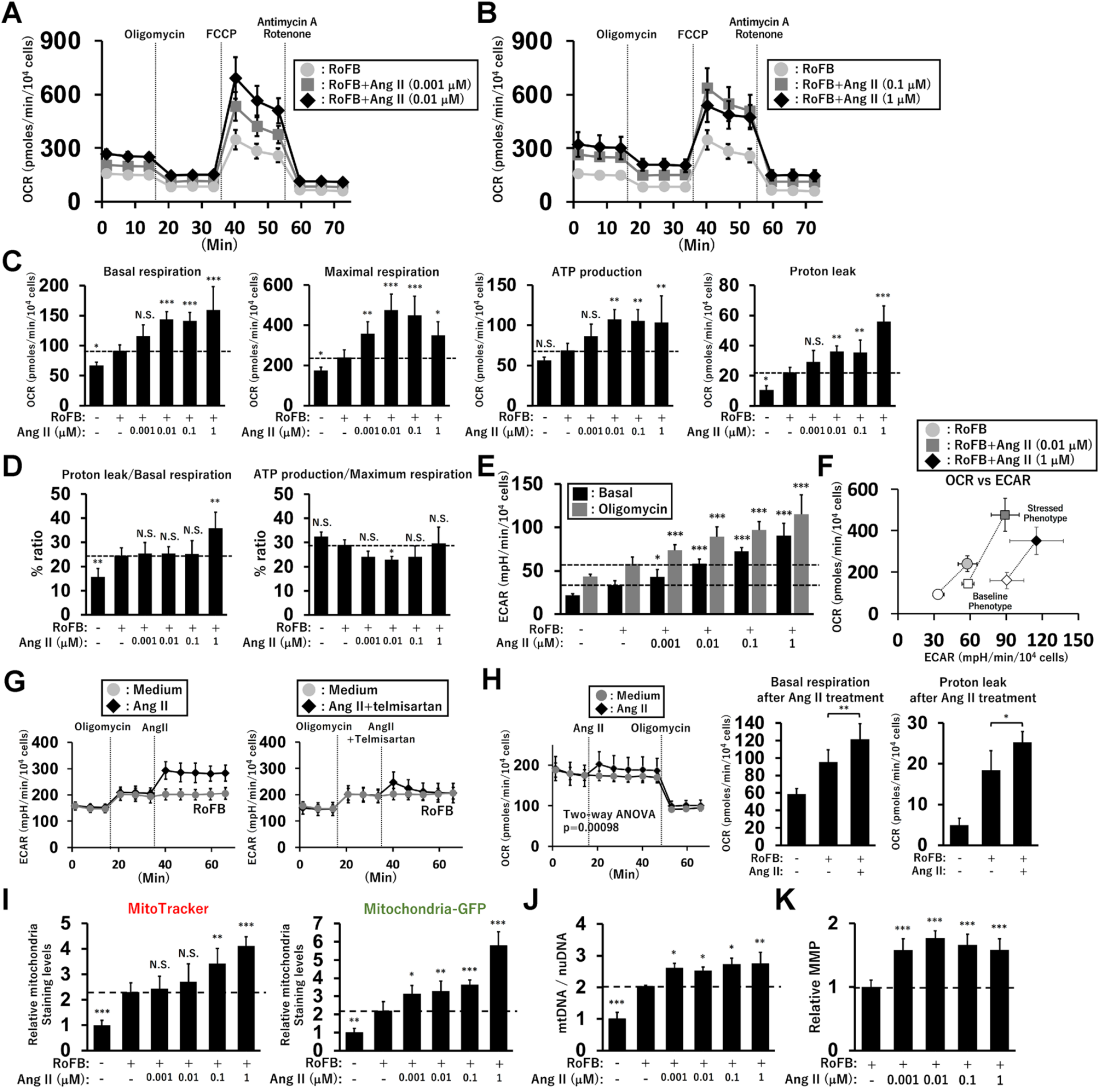
Elevation of mitochondrial oxygen consumption rate (OCR) and extracellular acidification rate (ECAR) by Ang II in ciBAs. (**A**) OCR was measured using Seahorse XFe96 extracellular flux analyser in control ciBAs converted by RoFB only (light grey circles), ciBAs continuously treated with Ang II at 0.001 μM (grey squares), and at 0.01 μM (black diamonds). (**B**) OCR was measured in the control ciBAs (light grey circles), ciBAs continuously treated with Ang II at 0.1 μM (grey squares), and at 1 μM (black diamonds). Mitochondrial respiration inhibitors, oligomycin, FCCP, and antimycin A/rotenone were sequentially added during the measurement as indicated. (**C**) OCR corresponding to basal respiration, maximal respiration, ATP production, and proton leak was calculated for comparison between the control and Ang II-treated ciBAs. (**D**) The ratios between OCR corresponding to proton leak and basal respiration, and between ATP production and maximum respiration were calculated. (**E**) ECAR in basal and oligomycin-treated conditions in ciBAs continuously treated with Ang II. (**F**) Energy phenotype profile in the control (light grey circles) and Ang II-treated ciBAs at 0.01 μM (grey squares) and 1 μM (black diamonds). OCR and ECAR are plotted under baseline (open circle, square, and diamond) and stressed (closed circle, square, and diamond) conditions. The stressed condition indicated the presence of oligomycin and FCCP. (**G**) Ang II (0.1 μM) and telmisartan (5 μM) were added to the control ciBAs during the measurement of ECAR as indicated. (**H**) OCR corresponding to basal respiration and proton leak was measured after the addition of Ang II (0.1 μM). Data represent mean ± SD (*n* = 6–8). *p*-values were determined using two-way ANOVA. (**I**) Mitochondria contents were measured by simultaneous labelling using MitoTracker and Mitochondria-GFP in ciBAs continuously treated with Ang II at various concentrations. (**J**) The relative ratio of mitochondrial DNA (mtDNA) to nuclear DNA (nuDNA) was determined by qPCR analysis in ciBAs continuously treated with Ang II. Data represent mean ± SD (*n* = 3). (**K**) Mitochondrial membrane potential (MMP) was evaluated by staining with the fluorescent dye, MT-1, in ciBAs continuously treated with Ang II. *p*-values were determined using one-way ANOVA with Tukey’s multiple comparison test: *p < 0.05, **p < 0.01, ***p < 0.001, N.S.; not significant.

Despite reduced lipolysis by Ang II, OCR was increased by continuous treatment with Ang II (Fig. 4A,B). The OCR increased from 0.001 μM and almost reached a plateau at concentrations greater than 0.01 μM. The OCR, corresponding to basal and maximal respiration, ATP production, and proton leak was elevated by Ang II treatment (Fig. 4C). The percent ratio of proton leak in basal respiration was only enhanced in ciBAs treated with Ang II at 1 μM, indicating that uncoupling efficiency was largely consistent in Ang II-treated ciBAs (Fig. 4D). The percent ratio of ATP production in maximum respiration was slightly reduced, indicating that the portion utilised by maximum respiratory capacity was almost unchanged by Ang II treatment. Notably, extracellular acidification rate (ECAR) strongly increased under both basal and oligomycin-treated conditions (Fig. 4E). Ang II-treated ciBAs were metabolically active and showed increase in both OCR and ECAR (Fig. 4F). ECAR immediately increased upon the addition of Ang II during measurement, while simultaneous treatment with telmisartan largely repressed this increase (Fig. 4G). Importantly, the addition of Ang II enhanced the OCR corresponding to both basal respiration and proton leak, compared with that in the untreated ciBAs (Fig. 4H). Mitochondrial staining with two different types of fluorescent probes indicated that Ang II enhanced the mitochondrial content in a dose-dependent manner (Fig. 4I and Supplementary Fig. S3B). The increased ratio of mitochondrial DNA to nuclear DNA indicated increased mitochondria in Ang II-treated ciBAs (Fig. 4J). Consistent with enhanced OCR, mitochondrial membrane potential (MMP) was elevated in Ang II-treated ciBAs (Fig. 4K and Supplementary Fig. S3C). These results indicated that Ang II robustly and rapidly promoted glucose utilisation through AGTR1, which contributes to coupled and uncoupled mitochondrial respiration in ciBAs.

**Figure 5:**
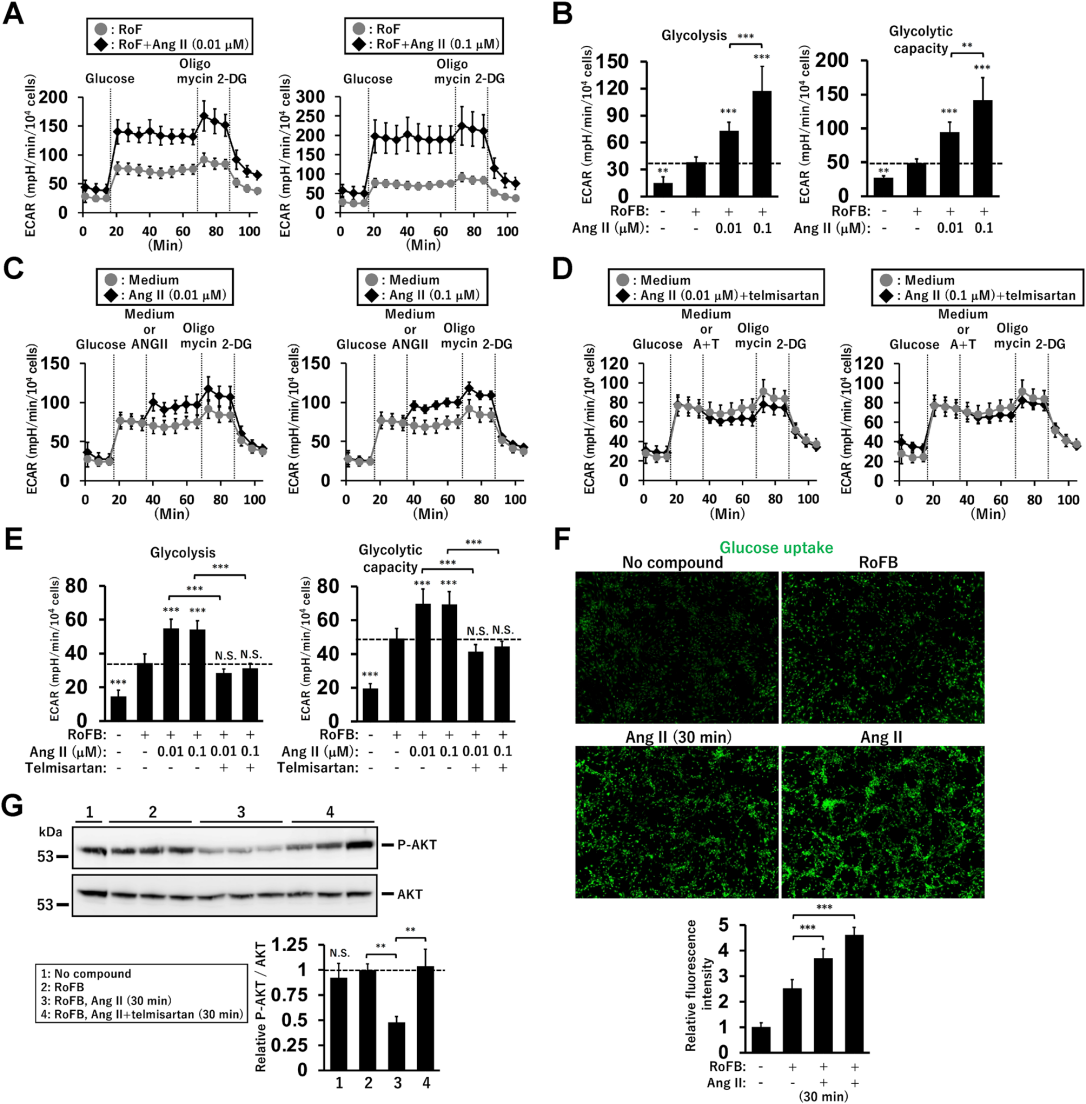
Activation of glycolysis rate and glucose uptake by Ang II in ciBAs. (**A**) ECAR was measured in the control (light grey circles) and ciBAs continuously treated with Ang II at concentrations of either 0.01 μM or 0.1 μM (black diamonds). Glucose, oligomycin, and 2-DG were sequentially added during the measurement, as indicated. (**B**) ECAR corresponding to glycolysis and glycolytic capacity was calculated in the Ang II-treated ciBAs. (**C**, **D**) Either Ang II or the combination of Ang II and telmisartan was added to the control ciBAs during the measurement, as indicated. (**E**) ECAR corresponding to glycolysis and glycolytic capacity was calculated in ciBAs transiently treated with either Ang II or the combination of Ang II and telmisartan. Data represent mean ± SD (*n* = 6–8). (**F**) Glucose uptake was evaluated by the fluorescent probe in ciBAs transiently (30 min) and continuously treated with 0.1 μM Ang II. The area of the staining was quantified using ImageJ software. (**G**) The phosphorylation of AKT was quantified by immunoblotting in ciBAs treated with either Ang II or the combination of Ang II and telmisartan for 30 min. The band intensities were measured by densitometry using ImageJ software. Data represent mean ± SD (*n* = 3). *p*-values were determined using one-way ANOVA with Tukey’s multiple comparison test: *p < 0.05, **p < 0.01, ***p < 0.001, N.S.; not significant.

To further characterise glucose metabolic processes activated by Ang II, a glycolysis stress assay was performed using glucose, oligomycin, and 2-deoxy-D-glucose (2-DG) in ciBAs cultured under glucose-free conditions. The addition of glucose increased glycolysis, while oligomycin maximised this rate by inhibiting mitochondrial ATP production. The continuous treatment with Ang II at 0.01 and 0.1 μM strongly enhanced ECAR compared to that in the untreated ciBAs (Fig. 5A). The ECAR, corresponding to glycolysis and glycolytic capacity, was elevated by Ang II treatment (Fig. 5B). The addition of Ang II to the control ciBAs during measurement immediately increased ECAR (Fig. 5C), which was inhibited by simultaneous treatment with telmisartan (Fig. 5D). Consistent with this observation, Ang II and telmisartan increased and inhibited glycolysis and glycolytic capacity, respectively (Fig. 5E). In addition, the OCR was enhanced upon treatment with Ang II, which was reversed by telmisartan treatment (Supplementary Fig. S4A,B). Glucose uptake, evaluated by a fluorescent glucose probe, was robustly increased by both transient and continuous treatment with Ang II (Fig. 5F). The phosphorylation of the serine/threonine kinase (AKT) in the insulin signalling pathway was reduced by both continuous and transient treatments with Ang II, which was reversed by telmisartan (Fig. 5G and Supplementary Fig. S5). These results support the conclusion that Ang II treatment coordinates cellular and mitochondrial energy metabolism by activating glycolysis and glucose uptake in ciBAs.

### Ang II treatment does not activate the transcription of glucose metabolic genes

**Figure 6:**
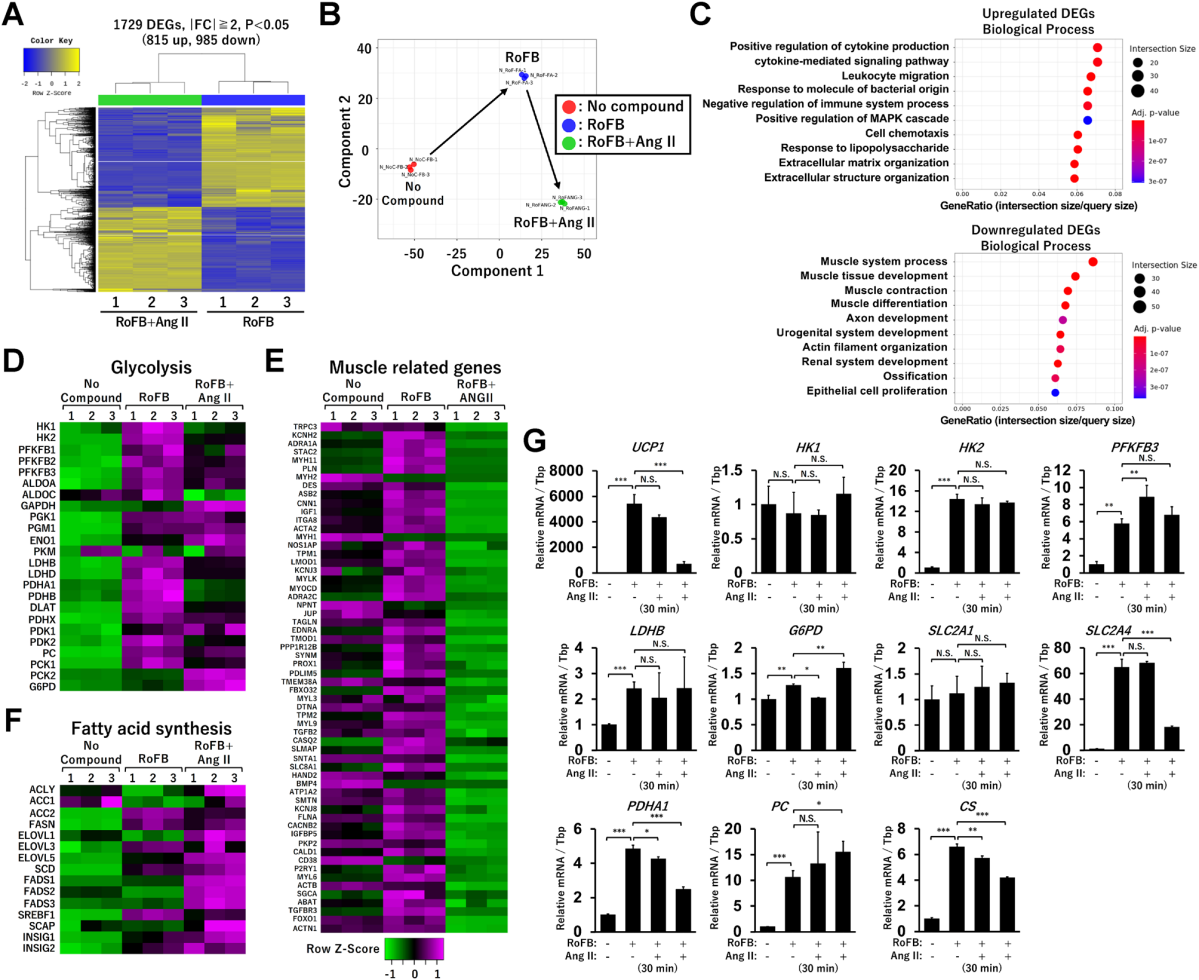
Transcriptome analysis in ciBAs continuously treated with Ang II. (**A**) Heat map and hierarchical clustering analysis representing 1,729 differentially expressed genes (DEGs) (|fold change (FC)|≥ 2, *p* < 0.05) between the control ciBAs (RoFB) and ciBAs continuously treated with Ang II (RoFB + Ang II) at 0.1 μM. (**B**) Multidimensional scaling analysis graphically indicates the similarity and variability of total expression patterns of the control fibroblast (NoC), RoFB, and RoFB + Ang II. (**C**) Gene ontology (GO) enrichment analysis was performed in the up- and down-regulated DEGs. The top 10 GO terms are represented in the category of biological process. (**D–F**) Heat maps represent transcriptional profiles in functional groups, such as glycolysis, muscle related genes, and fatty acid synthesis. The colour scale shows z-scored fragments per kilobase of transcript per million mapped sequence reads (FPKM) representing mRNA levels of each gene in green (lower expression) and magenta (higher expression). (**G**) The expression of glucose metabolic genes was quantified by qRT-PCR analysis. Data represent mean ± SD (*n* = 3). *p*-values were determined using one-way ANOVA with Tukey’s multiple comparison test: *p < 0.05, **p < 0.01, ***p < 0.001, N.S.; not significant.

To address how Ang II treatment alters metabolic gene expression, a genome-wide transcriptome analysis was performed. A comparison of the RNA-Seq data between control (RoFB) and AngII-treated ciBAs (RoFB + Ang II) revealed 815 upregulated and 985 downregulated differentially expressed genes (DEGs) (Fig. 6A). The smear and volcano plots showed that DEGs with over two-fold changes (FCs) were appropriately distributed with widespread counts per million (CPM) and *p*-values (Supplementary Fig. S6A). Venn diagrams show that 787 upregulated and 575 downregulated DEGs overlapped (Supplementary Fig. S6B). Multidimensional scaling analysis visually suggested that Ang II treatment affected the transcriptome of ciBAs (Fig. 6B). However, gene ontology (GO) enrichment analysis showed that the upregulated DEGs were not directly associated with glucose or lipid metabolism (Fig. 6C). Ang II treatment downregulated a series of muscle-related genes. Heat maps represented that Ang II treatment did not consistently increase the expression of glucose metabolism-related genes (Fig. 6D), whereas many muscle-related genes were repressed (Fig. 6E and Supplementary Table S1). Although several metabolic genes involved in the tricarboxylic acid (TCA) cycle and fatty acid β-oxidation were rather reduced (Supplementary Fig. S6C), the expression of genes involved in fatty acid synthesis was slightly increased by Ang II treatment (Fig. 6F). Quantitative real-time polymerase chain reaction (qRT-PCR) analysis confirmed that the expression of glucose metabolic genes, such as *HK1*, *HK2*, *PFKFB3*, *PDHA1*, *LDHB*, *G6PD*, *SLC2A1*, *SLC2A4*, *PDHA1*, *PC*, and *CS*, was not robustly increased by both transient and continuous treatment with Ang II (Fig. 6G). These results indicated that the activation of glycolysis by Ang II was unlikely to be due to transcriptional changes in metabolic genes.

## Discussion

**Figure 7:**
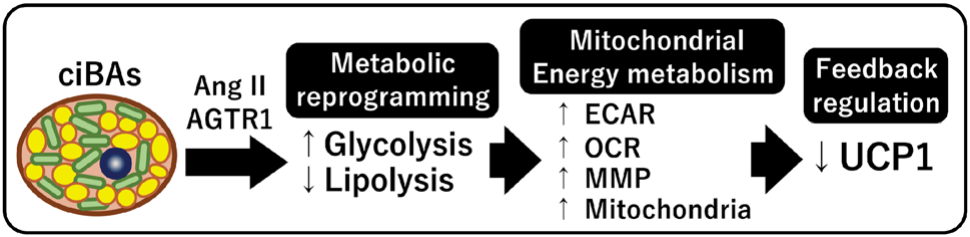
A schematic illustration of Ang II effects on metabolic reprogramming and mitochondrial energy metabolism in ciBAs. Ang II rapidly and robustly promoted glycolysis in ciBAs through AGTR1, while Ang II repressed triglyceride lipolysis. Despite repressed lipolysis, Ang II elevated ECAR, OCR, MMP, and mitochondria content. Consistent with our previous observation, *UCP1* transcription was downregulated under elevated mitochondrial energy status by Ang II likely to avoid excess uncoupled respiration.

In this study, we revealed that Ang II treatment affected thermogenic functions and cellular energy metabolism in ciBAs (Fig. 7). Ang II treatment repressed the transcription of *UCP1* and *CIDEA* as well as triglyceride lipolysis and storage. Reduced lipolysis by Ang II was associated with the downregulation of ATGL, CEBPA, and HSL phosphorylation. Despite this, Ang II-treated ciBAs showed increased mitochondrial OCR and MMP along with elevated mitochondrial contents. Notably, Ang II treatment rapidly and highly activated glycolysis and glucose uptake, which likely promoted metabolic reprogramming from lipolysis to glycolysis in ciBAs. Importantly, the activation of glycolysis was associated with an increase in coupled and uncoupled mitochondrial respiration. The effects of Ang II on glucose and lipid metabolism were exerted through the Ang II/AGTR1 axis, and treatment with telmisartan largely abolished these effects. We previously proposed a feedback regulation between mitochondrial energy status and thermogenic *UCP1* transcription in ciBAs^40,41^. MMP induced by enriched free fatty acids, mimicking obese conditions, downregulated *UCP1* transcription likely to avoid excess heat generation. In contrast, the depletion of free fatty acids induced *UCP1* expression likely to compensate for uncoupled proton leak under low MMP conditions. The feedback regulation is considered beneficial for coordinating the thermogenic proton leak activity mediated by UCP1 in brown adipocytes. Our study also found that the Ang II-activated mitochondrial energy status led to the repression of *UCP1* transcription, similar to the feedback regulation described above. These results may partially explain the obesity-associated repression of *UCP1* expression under the hyperactivation of the RAS during obesity^19,41^.

Our previous transcriptome analysis revealed that a specific set of the RAS components, including AGT and AGTR1, was expressed in multiple human adipocyte models derived from HDFs, hTERT A41hBAT-SVF, and AdMSCs (Fig. 1). Treatment with telmisartan under exogenous Ang II-free conditions did not influence the expression of *UCP1* and *CIDEA*, implying that Ang II was not locally produced from AGT in ciBAs. Ang II is deactivated by ACE2 and converted into angiotensin 1-7 peptide, Ang (1-7), acting as an agonist of MAS1. The counter-regulatory ACE2/Ang (1-7)/MAS1 axis is known to have beneficial effects on diet-induced weight gain, adipocyte browning, thermogenesis, and metabolic complications via the activation of AKT and inhibition of the ERK MAPK signalling pathway^14,42^. Mice with whole-body knockout of either ACE2 or MAS1 displayed metabolic abnormalities, whereas either overexpression of ACE2 or continuous infusion of Ang (1-7) peptide improved blood glucose, lipid, and energy expenditure, along with enhanced adipocyte browning in WAT^43^. Another recent study indicated that hamster BAT was activated by the third ventricular injection of Ang (1-7) peptide through the Mas1 receptor in brain regions^44^. In this study, the treatment with Ang (1-7) had little effect on the expression levels of *UCP1*, *CIDEA*, and *FABP4*, suggesting that the counter-regulatory axis is not functional in ciBAs. This was consistent with the observation that MAS1 expression was negligible in ciBAs and the other models of human adipocytes. These results indicated that the classical Ang II/AGTR1 axis may be the major RAS pathway in human brown adipocytes.

The short-term treatment with Ang II inhibited βAR-mediated induction of *UCP1* expression, lipolysis, and cAMP levels (Supplementary Fig. S7A). Previous studies have reported that the Gαi protein is one of the effectors of AGTR1^17^. Gαi reduces cAMP levels by inhibiting the adenylyl cyclase activity in response to Ang II treatment, suggesting that Ang II effects partially overlapped with the thermogenesis pathway^45^. This is further supported by the observation that continuous treatment with Ang II decreased the phosphorylation of HSL and CREB as downstream events in the cAMP signalling pathway (Supplementary Fig. S7B)^41^. However, transient treatment with Ang II enhanced the phosphorylation of CREB, but not of HSL. The canonical signalling of the AGTR1 receptor also couples to the Gαq protein to activate phospholipase C (PLC), resulting in the activation of PKC by the increase of Ca^2+^ concentrations and diacylglycerol (DAG) (Supplementary Fig. S7A)^46,47^. The potent PKC inhibitor, Go6983, robustly reduced CREB phosphorylation by Ang II, suggesting that AGTR1 positively and negatively contributes to the phosphorylation of CREB through Gαq and Gαi, respectively. In addition, our results indicated that the PKA pathway was not closely associated with Ang II-mediated CREB phosphorylation. Similar to other reports^15,17^, this study indicated that the phosphorylation of p38 and ERK in the MAPK signalling pathway was increased by both transient and continuous treatment with Ang II. The results obtained using each inhibitor indicated that they both partially contributed to Ang II-induced CREB phosphorylation.

It has been reported that Ang II is involved in the pathogenesis of insulin resistance and cardiovascular diseases^47,48^. Adipose tissue-specific overexpression of AGT or Ang II infusion caused systemic insulin resistance^49^. However, Ang II did not alter the binding and activity of the insulin receptor^50,51^. Mechanistically, Ang II negatively regulates the insulin signalling cascade, including the tyrosine phosphorylation of the insulin receptor and insulin receptor substrates, phosphatidylinositol 3-kinase (PI3K), and AKT^52,53^. This study found that Ang II robustly enhanced glycolytic flux and glucose uptake in ciBAs. AKT phosphorylation was repressed by Ang II through AGTR1, implying that Ang II-activated glycolysis is unlikely to occur through the activation of the insulin signalling pathway. In previous studies, Ang II inhibited adipocyte lipolysis by approximately 20–25% in human subjects in an AGTR1-dependent manner^54,55^. This suggests that metabolic reprogramming from lipolysis to glycolysis is promoted by Ang II treatment. Ang II-activated glycolysis contributes to the elevation of mitochondrial OCR, suggesting that glycolytic pyruvate may be used for the anaplerosis of the TCA cycle. However, transcriptome analysis of Ang II-treated ciBAs revealed that the transcription of glucose and lipid metabolic genes remained largely unchanged, despite the activation of glycolysis. Although a series of muscle-related genes were consistently repressed by Ang II, their association with either glycolysis or *UCP1* expression remains unclear. Rapid response to Ang II appears to be mediated by post-translational and/or post-transcriptional changes that regulate the glycolytic pathway through AGTR1. Further research is required to clarify the precise molecular mechanisms underlying the activation of glycolysis by Ang II.

To date, the regulation of glycolysis and glucose uptake by Ang II has not been extensively examined in brown adipocytes. Therefore, this study provides additional insights into this mechanism and its association with the thermogenic functions of human brown adipocytes. The elevated mitochondrial energy status through the Ang II/AGTR1 axis may be beneficial for the efficient consumption of blood glucose for heat production in brown adipocytes. Although angiotensin-converting enzyme inhibitors and Ang II receptor blockers are expected to prevent type 2 diabetes and obesity-associated metabolic disorders^47,48,56^, this study implies that they might affect some aspects of systemic glucose metabolism through brown adipocytes and other metabolic cells. Importantly, cold exposure potentially elevates circulating Ang II and NE levels along with the SNS activation^30–32^. This study suggests the possible contribution of Ang II to adaptive thermogenesis in human brown adipocytes. We previously reported that treatment with capsaicin directly activated UCP1 expression along with other browning features in ciBAs^57^. Thus, the thermogenic functions of ciBAs can be distinctively regulated by bioactive molecules through cell surface receptors, such as AGTR1 and transient receptor potential vanilloid 1 (TRPV1). Insights obtained from ciBAs are supportive of uncovering the molecular mechanism of the action targeting human brown adipocytes.

## Methods

### Cell culture

Fibroblasts derived from a human subject aged 38 years (HDF38) (PromoCell, Heidelberg, Germany) were used for this study^39^. The commercial human cells have been approved for in vitro research use only. All experimental procedures for cell cultures were conducted in accordance with the general guidelines of the Kyoto Prefectural University of Medicine. Approximately 1.5 × 10^5^ cells were seeded in a 35-mm dish with high-glucose Dulbecco's modified Eagle medium (DMEM) (11995-065, Gibco, MA, USA) supplemented with 10% fetal bovine serum (FBS) (HyClone, UT, USA) and penicillin/streptomycin (Gibco). After reaching 80–90% confluence, the medium was changed to start the direct conversion into ciBAs with the serum-free brown adipogenic medium (SFBAM), either with or without the chemical cocktail RoFB, which consisted of 1 μM rosiglitazone (FUJIFILM Wako), 7.5 μM forskolin (FUJIFILM Wako), and 20 ng/mL human recombinant BMP7 (FUJIFILM Wako) for 3 weeks, as reported previously^38^. Angiotensin II (015-27911, FUJIFILM Wako) was added in combination with RoFB during conversion at the indicated concentrations. The medium was changed every 3 days. Angiotensin (1–7) peptide (A9202, Sigma-Aldrich, MO, USA), telmisartan (205-20691, FUJIFILM Wako), MLN-4760 (31,327, Cayman Chemical, MI, USA), isoproterenol (I0260, Tokyo Chemical Industry, Tokyo, Japan), norepinephrine (A0906, Tokyo Chemical Industry), PD98059 (FUJIFILM Wako), SB202190 (FUJIFILM Wako), Go6983 (FUJIFILM Wako), and KN-62 (Cayman Chemical) were used to treat ciBAs as indicated.

### qRT-PCR

Total RNA was extracted from control fibroblasts and ciBAs cultured under each experimental condition using the FastGene RNA Basic Kit (Nippon Genetics, Tokyo, Japan). Reverse transcription was conducted using ReverTra Ace qPCR RT Master Mix with gDNA Remover (TOYOBO, Osaka, Japan). qRT-PCR analysis was performed using Power SYBR Green PCR Master Mix (Applied Biosystems, MA, USA). The reactions were carried out in triplicate under the following conditions: 10 min at 95 °C, followed by 40 cycles of 15 s at 95 °C and 60 s at 60 °C. All results were normalised to *TBP* mRNA levels. Primer sequences used for qRT-PCR are listed in Supplementary Table S2. Unless otherwise indicated, the average of the three biological replicates was calculated.

### Immunoblotting

Total protein was extracted from control fibroblasts, ciBAs, and ciBAs treated with Ang II and/or small molecules using RIPA buffer (FUJIFILM Wako) supplemented with a phosphatase inhibitor cocktail (FUJIFILM Wako) and protease inhibitor cocktail (FUJIFILM Wako). The extracted proteins were subjected to sodium dodecyl sulfate–polyacrylamide gel electrophoresis using a 10% gel concentration and transferred to a polyvinylidene fluoride membrane (Thermo Fisher Scientific, MA, USA). The membranes were blocked with 3% skim milk followed by incubation with antibodies against UCP1 (MAB6158, R&D systems, MN, USA), ATGL (#2138, Cell Signaling Technology, MA, USA), CEBPA (#8178, Cell Signaling Technology), β-Actin (A5316, Sigma-Aldrich), P-HSL (#18381, Cell Signaling Technology), HSL (#4107, Cell Signaling Technology), P-CREB (#9198, Cell Signaling Technology), CREB (#9197, Cell Signaling Technology), P-p38 (#4511, Cell Signaling Technology), p38 (#8690, Cell Signaling Technology), P-ERK1/2 (#4370, Cell Signaling Technology), ERK1/2 (#4695, Cell Signaling Technology), P-AKT (#4060, Cell Signaling Technology), and AKT (#4691, Cell Signaling Technology) overnight at 4 °C or for 2 h at room temperature. Membranes were then incubated with horseradish peroxidase (HRP)-conjugated anti-rabbit or anti-mouse secondary antibodies (Cell Signaling Technology) for 1 h at room temperature. Immunoreactive bands were detected using Immobilon Western Chemiluminescent HRP Substrate (Merck Millipore, Darmstadt, Germany). The intensity of each band was quantified via densitometry using ImageJ software (version 1.52; National Institutes of Health, Bethesda, MD, USA). Experiments were independently performed at least twice.

### Immunocytochemistry

The cells were preincubated with 1 µM Lipi-Red (Dojindo, Kumamoto, Japan) for 30 min at 37 °C in 5% CO_2_, according to the manufacturer’s instructions. Subsequently, the cells were fixed with 4% paraformaldehyde for 10 min. After washing with phosphate-buffered saline (PBS), the cells were incubated in PBS containing 0.1% Triton X-100 for 5 min. Then, they were blocked with PBS containing 3% skim milk for 1 h at room temperature and incubated again with UCP1 antibody (ab10983, Abcam, Cambridge, UK) at 1/2000 dilution overnight at 4 °C. After washing with PBS, the cells were incubated with Alexa Fluor 488 donkey anti-rabbit IgG antibody (Invitrogen, CA, USA) for 1 h at room temperature. Subsequently, cell nuclei were stained with 4′,6-diamidino-2-phenylindole (DAPI) solution (Dojindo). All images were obtained using a BZ-X710-All-in-One Fluorescence Microscope (Keyence, Osaka, Japan) with a 20X objective lens (CFI Plan Fluor 20X, Nikon, Tokyo, Japan). All scale bars represent 200 μm. The areas of Lipi-Red and UCP1 staining were quantified using ImageJ software from at least five different optical sections.

### Measurement of mitochondria contents and membrane potentials

For MMP dependent- and independent-staining, ciBAs were stained by MitoTracker® Red CM-H_2_XRos (Thermo Fisher Scientific, DE, USA) and CellLight® Mitochondria-GFP, BacMam 2.0 (C10600, Invitrogen), respectively. In brief, the cells were pre-incubated with CellLight reagent for 1 d and with MitoTracker for 30 min at 37 °C in a 5% CO_2_ before the cells were fixed with 4% paraformaldehyde for 10 min. MMPs in control fibroblasts and ciBAs were evaluated using the MT-1 MitoMP Detection kit (MT13, Dojindo). The cells were treated with the MT-1 dye for 30 min at 37 °C in 5% CO_2_ incubator, in accordance with the instructions. Then, the cells were fixed with 4% paraformaldehyde for 10 min. All the images were captured using a BZ-X710-All-in-One Fluorescence Microscope. The area of staining was quantified from at least five different optical sections using ImageJ software.

### Measurement of glucose uptake

To quantify glucose uptake, each ciBA was treated with a fluorescent glucose uptake probe (Glucose Uptake Assay Kit-Green, 341-09821, Dojindo) according to the manufacturer’s instructions. Briefly, the cells were washed twice with DMEM without glucose or serum. After washing, the probe was incubated for 15 min at 37 °C in 5% CO_2_. After washing twice with an ice-cold solution, the cells were incubated at room temperature for 5 min in the same solution. After replacing the solution once, images were captured using a BZ-X710-All-in-One Fluorescence Microscope. The staining was quantified as described above.

### Quantification of mitochondrial DNA

Total genomic DNA was extracted from control and Ang II-treated ciBAs using NucleoSpin Tissue (TaKaRa Bio). Mitochondrial DNA (mtDNA) copy numbers were measured by qPCR using the Power SYBR Green PCR Master Mix. Each mtDNA amount was normalised to the corresponding nuclear DNA (nuDNA) level. Primer sequences used for the quantification of mitochondrial and nuclear DNA were as follows: mtDNA-Fwd, ACACCCTCCTAGCCTTACTAC; mtDNA-Rev, GATATAGGGTCGAAGCCGC; nuDNA-Fwd, AGGGTATCTGGGCTCTGG; and nuDNA-Rev, GGCTGAAAAGCTCCCGATTAT^58^. The average of three biological replicates was calculated.

### Measurement of OCR and ECAR

To measure the mitochondrial OCR, ciBAs were prepared in a 96-well plate under different experimental conditions for 3 weeks. Before measurement, cells were washed and incubated with non-buffered DMEM supplemented with 25 mM glucose, 2 mM glutamine, and 1 mM pyruvate at 37 °C in a non-CO_2_ incubator for 1 h. The OCR was measured using the Seahorse XF96 extracellular flux analyer (Seahorse Bioscience Inc., MA, USA) according to the manufacturer’s instructions. During the analysis, oligomycin, carbonyl cyanide 4-(trifluoromethoxy)phenylhydrazone (FCCP), and antimycin A/rotenone were added to each well using an injection apparatus at final concentrations of 2, 0.5, and 0.5 μM, respectively. The OCR corresponding to each mitochondrial parameter was determined by subtracting the antimycin A/rotenone-insensitive OCR values from other OCR values. To evaluate the rapid effects of Ang II on the OCR and ECAR, Ang II was added to each well via an injection apparatus during measurement. For measurement of glycolysis flux, the cells were incubated in non-buffered DMEM without glucose and pyruvate for 1 h at 37 °C in a non-CO_2_ incubator. ECAR was measured using the Flux Analyzer by adding glucose (Sigma-Aldrich), oligomycin (Sigma-Aldrich), and 2-deoxy-D-glycose (Tokyo Chemical Industry) via an injection apparatus during the measurement at final concentrations of 10 mM, 5 μM, and 50 mM, respectively. The ECAR corresponding to glycolysis and glycolytic capacity was determined by subtracting the 2-DG-insensitive ECAR values from the other ECAR values before and after oligomycin treatment, respectively.

### Measurement of glycerol, triglycerides, cAMP, AGT, and cytotoxicity

Cell culture supernatants and extracted cellular lysates were collected from the control fibroblasts, ciBAs, and ciBAs treated with Ang II, telmisartan, or Iso. The amounts of free glycerol and triglycerides were measured using Free Glycerol Assay Kit (ab65337, Abcam) and Triglyceride Assay kit (Ab65336, Abcam), respectively, according to the manufacturer’s instructions. cAMP levels in each ciBA sample were measured using a competitive ELISA method (cAMP Assay Kit, ab65355, Abcam). The secretion of AGT from ciBAs into the supernatant was quantified using a sandwich ELISA (Human Total Angiotensinogen Assay Kit, Immuno-Biological Laboratories, Gunma, Japan). To evaluate the cytotoxicity of Ang II treatment, the activity of lactate dehydrogenase (LDH) released into the culture supernatants was measured using Cytotoxicity LDH Assay Kit-WST (Dojindo) according to the manufacturer’s instructions. Glycerol, triglycerides, cAMP, and AGT levels were normalised to the total amounts of protein in each cell culture. All the experiments were performed in triplicates. Experiments were independently performed at least twice.

### RNA sequencing (RNA-Seq) analysis

RNA-Seq results for control fibroblasts (NoC) and ciBAs (RoFB) have been previously reported^39^. In this study, total RNA was prepared from 0.1 μM Ang II-treated ciBAs (RoFB + Ang II) derived from HDF38. The RNA integrity number values were more than 9 for all RNA samples extracted using the FastGene RNA Premium Kit (Nippon Genetics, Tokyo, Japan). The library was prepared using the TruSeq stranded mRNA LT Sample Prep Kit (Illumina, San Diego, CA, USA), following the manufacturer’s low-sample protocol. Paired-end sequencing (100 bp) was performed using the NovaSeq 6000 System (Illumina). Trimmed reads were mapped to a reference genome (NCBI GRCh37) using HISAT2. After the read mapping, StringTie was used for transcript assembly. After the assembly, the abundance of genes/transcripts was calculated from read counts and normalised as fragments per kilobase of transcript per million mapped sequence reads (FPKM). For the identification of DEGs, statistical analysis was performed using FC and an exact test using edgeR per comparison pair. Significant results satisfying the conditions of |FC|≥ 2 and an exact test *p*-value < 0.05 were selected. If more than one read count value was zero, it was excluded from the analysis. Heat maps were generated using Heatmapper (http://www.heatmapper.ca/)^59^. Hierarchical clustering analysis was performed based on the Euclidean distance. Each row represents a gene, and each column represents the z-scored FPKM of each sample. The green and magenta gradients represent lower and higher gene expression, respectively. GO enrichment analysis was performed using DAVID Bioinformatics Resources 6.8 (https://david.ncifcrf.gov/)^60^.

### Statistical analyses

All the results are presented as the mean ± standard deviation (SD). Differences between two independent groups were evaluated by two-tailed Student’s t-test using the Excel (Microsoft, WA, USA) program. Differences between multiple independent groups were analysed by one-way ANOVA or two-way ANOVA with Tukey’s multiple comparison test, unless otherwise stated in the figure legends, using an R-based statistical software, EZR version 1.65 (Saitama Medical Center, Jichi Medical University, Saitama, Japan)^61^. Statistical significance was defined as *p* < 0.05.

### Supplementary Information

Supplementary Information.

## Data Availability

The RNA-sequencing data have been deposited in the DNA Data Bank of Japan (DDBJ) Sequenced Read Archive (https://www.ddbj.nig.ac.jp/dra/index-e.html) under the accession number DRR530882.
