# Supplementary material for: Angiotensin II participates in mitochondrial thermogenic functions via the activation of glycolysis in chemically induced human brown adipocytes
Source: Sci Rep. 2024 May 11;14:10789. doi: 10.1038/s41598-024-61774-0 (PMC11088625; doi:10.1038/s41598-024-61774-0)
Supplement: Supplementary file 1 — Supplementary Information. [file 41598_2024_61774_MOESM1_ESM.pdf]

Angiotensin II participates in mitochondrial thermogenic functions via the activation of glycolysis in chemically induced human brown adipocytes.

Yukimasa Takeda[1*], Toshikazu Yoshikawa[2,3], and Ping Dai[1*]

[1]Department of Cellular Regenerative Medicine, Graduate School of Medical Science, Kyoto Prefectural University of Medicine, 465 Kajii-cho, Kawaramachi-Hirokoji, Kamigyo-ku, Kyoto 602-8566, Japan

[2]Louis Pasteur Center for Medical Research, 103-5 Tanaka-Monzen-cho, Sakyo-ku, Kyoto 606-8225, Japan

[3]Department of Molecular Gastroenterology and Hepatology, Graduate School of Medical Science, Kyoto Prefectural University of Medicine, 465 Kajii-cho, Kawaramachi-Hirokoji, Kamigyo-ku, Kyoto 602-8566, Japan

Supplementary Information

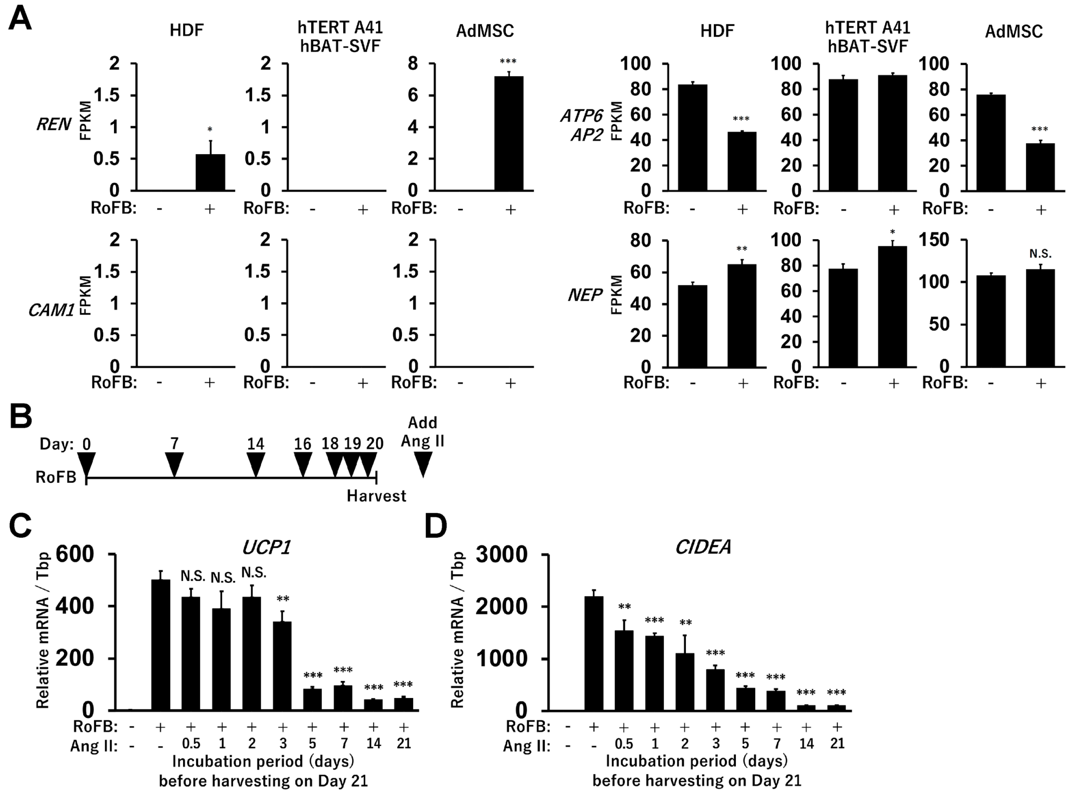

Supplementary Figure S1. Ang II effects on the transcription of the RAS components and *UCP1* in ciBAs. (**A**) Our previous RNA-Seq analyses provide the mRNA level of the RAS components, such as *REN*, *CAM1*, *ATP6AP2*, and *NEP* between the controls and adipocytes converted from HDFs, hTERT A41hBAT-SVF, and AdMSCs. Statistical: two-tailed Student's t-tests. (**B**) The illustration represents the timing of the addition of Ang II at 0.1 μM after the conversion of HDFs into ciBAs with the chemical cocktail, RoFB, on day 0. (**C,D**) The expression of *UCP1* and *CIDEA* was measured by qRT-PCR in ciBAs treated with Ang II for different incubation periods (days) before harvesting the cells on day 21. Data represent mean ± SD (n = 3). P values were determined using one-way ANOVA with Tukey's multiple comparison tests: ** $p < 0.01$, *** $p < 0.001$, N.S.; not significant.

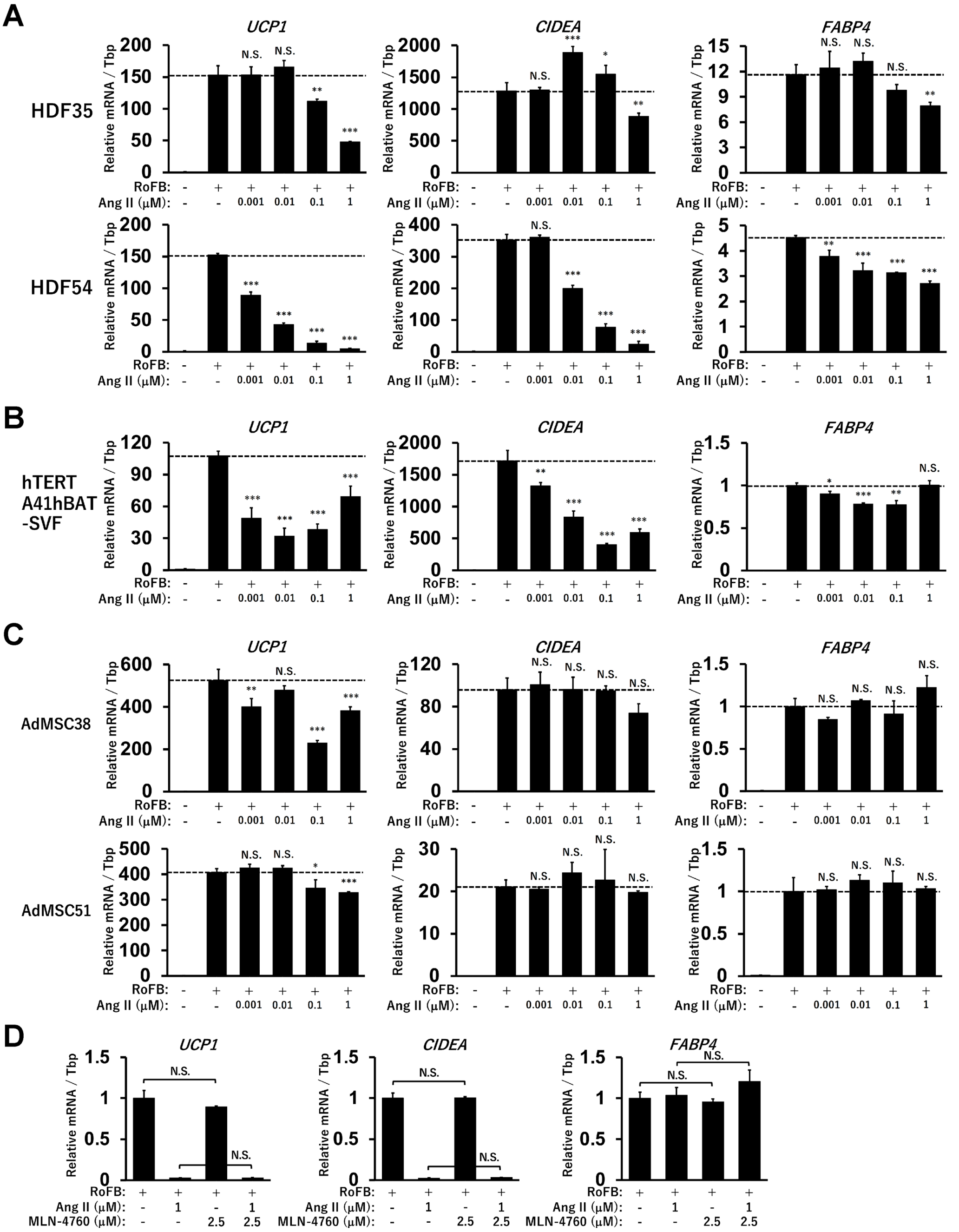

Supplementary Figure S2. Confirmation of Ang II effects on *UCP1* and *CIDEA* expression in ciBAs derived from other lines of HDFs (HDF35 and HDF54) (**A**), the adipocytes derived from hTERT-A41hBAT SVF (**B**) and AdMSCs (AdMSC38 and AdMSC51) (**C**). (**D**) The expression was quantified in ciBAs continuously treated with Ang II and MLN-4760, a potent ACE2 inhibitor, as indicated. Data represent mean ± SD (n = 3). P values were determined using one-way ANOVA with Tukey's multiple comparison tests: * $p < 0.05$, ** $p < 0.01$, *** $p < 0.001$, N.S.; not significant.

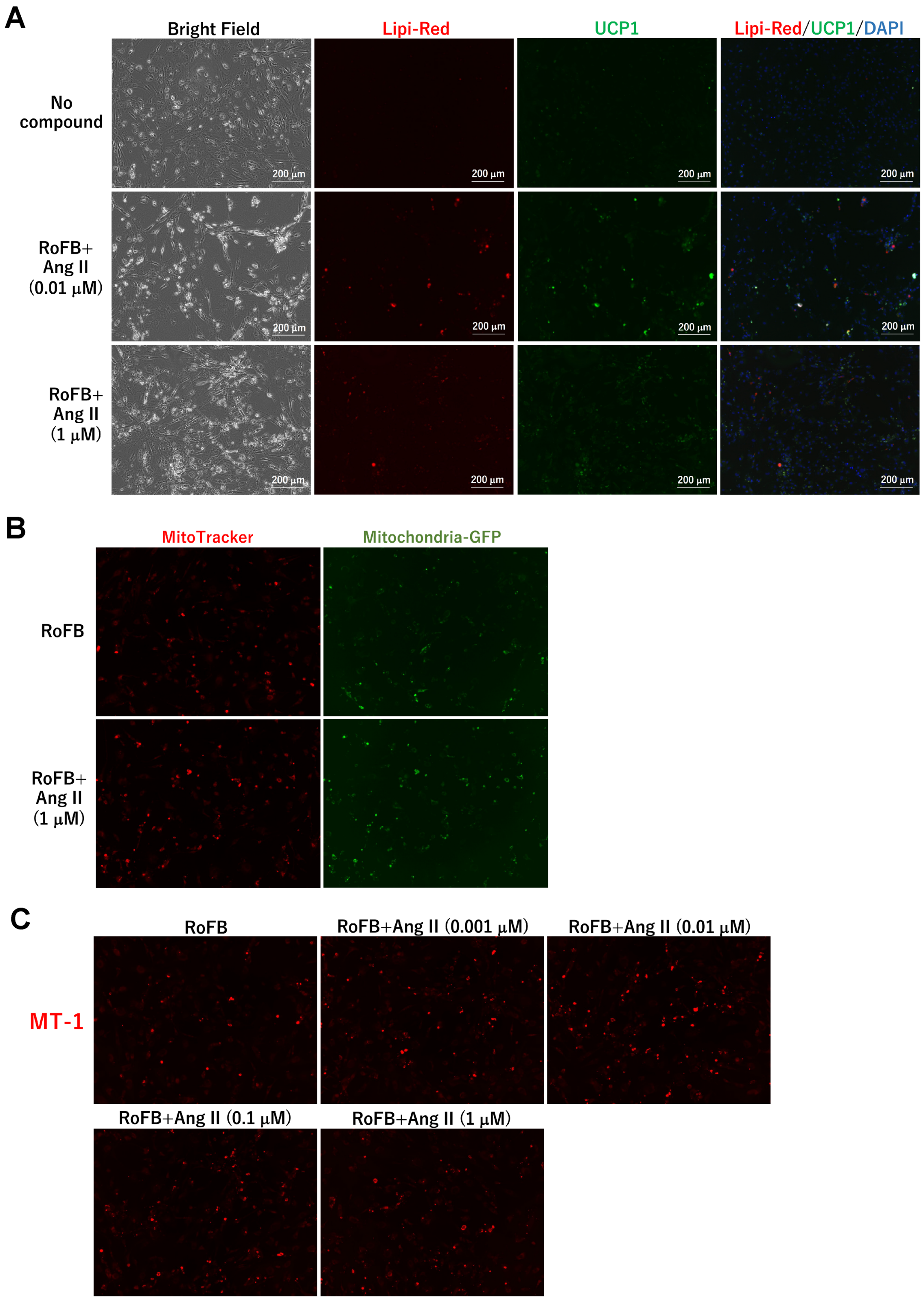

Supplementary Figure S3. Fluorescence analyses in ciBAs. (**A**) Representative images of bright field, Lipi-Red staining (red), UCP1 protein (green), and merged image in

control fibroblasts and ciBAs continuously treated with Ang II at the concentration of 0.01 μM and 1 μM. The nuclei were visualised by DAPI (blue). Scale bars represent 200 μm. (**B**) Mitochondria were stained by simultaneous labelling by MitoTracker and GFP localized in mitochondria (Mitochondria-GFP) in the control ciBAs and ciBAs continuously treated with Ang II at 1 μM. (**C**) Mitochondrial membrane potential (MMP) was stained by the fluorescent dye, MT-1, in the control ciBAs and ciBAs continuously treated with Ang II at various concentrations.

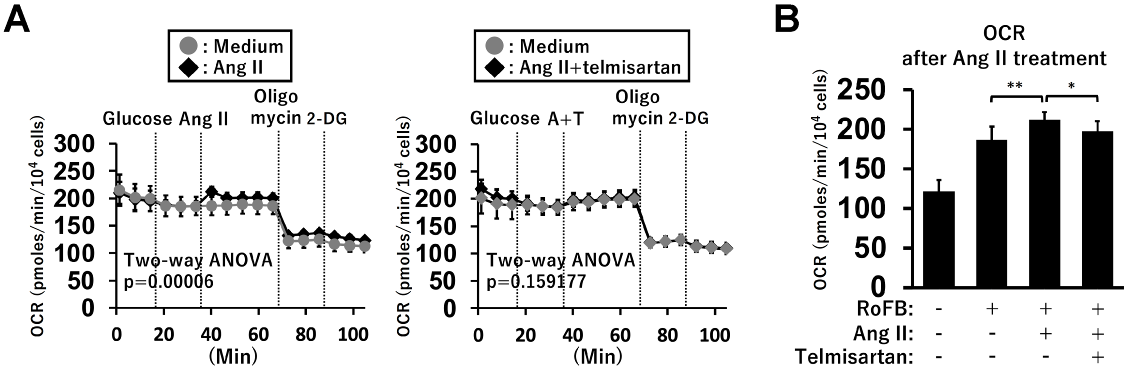

Supplementary Figure S4. Ang II effects on mitochondrial OCR. (**A**) OCR was measured in the control ciBAs (light grey circles) transiently treated with either Ang II at 0.1 μM or the combination of Ang II and telmisartan at 5 μM (black diamonds) during measurement. Glucose, oligomycin, and 2-deoxyglucose (2-DG) were sequentially added during measurement, as indicated. *P* values were determined using two-way ANOVA. (**B**) The change of OCR was compared in the ciBAs after the treatment with Ang II. Data represent mean ± SD (n = 6-8). *P* values were determined using one-way ANOVA with Tukey's multiple comparison tests: * $p < 0.05$, ** $p < 0.01$, *** $p < 0.001$, N.S.; not significant.

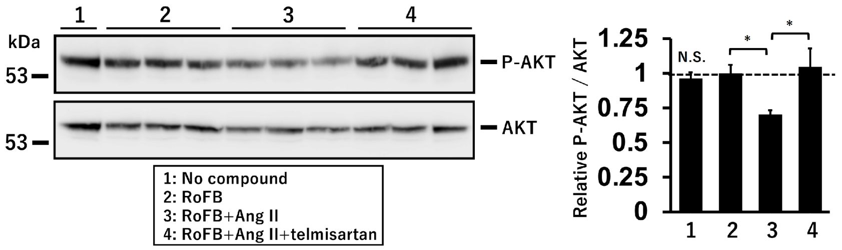

Supplementary Figure S5. AKT phosphorylation was quantified by immunoblotting in ciBAs continuously treated with either Ang II or the combination of Ang II and telmisartan. The band intensities were measured by densitometry using ImageJ software. *P* values were determined using one-way ANOVA with Tukey's multiple comparison tests: * $p < 0.05$, ** $p < 0.01$, N.S.; not significant.

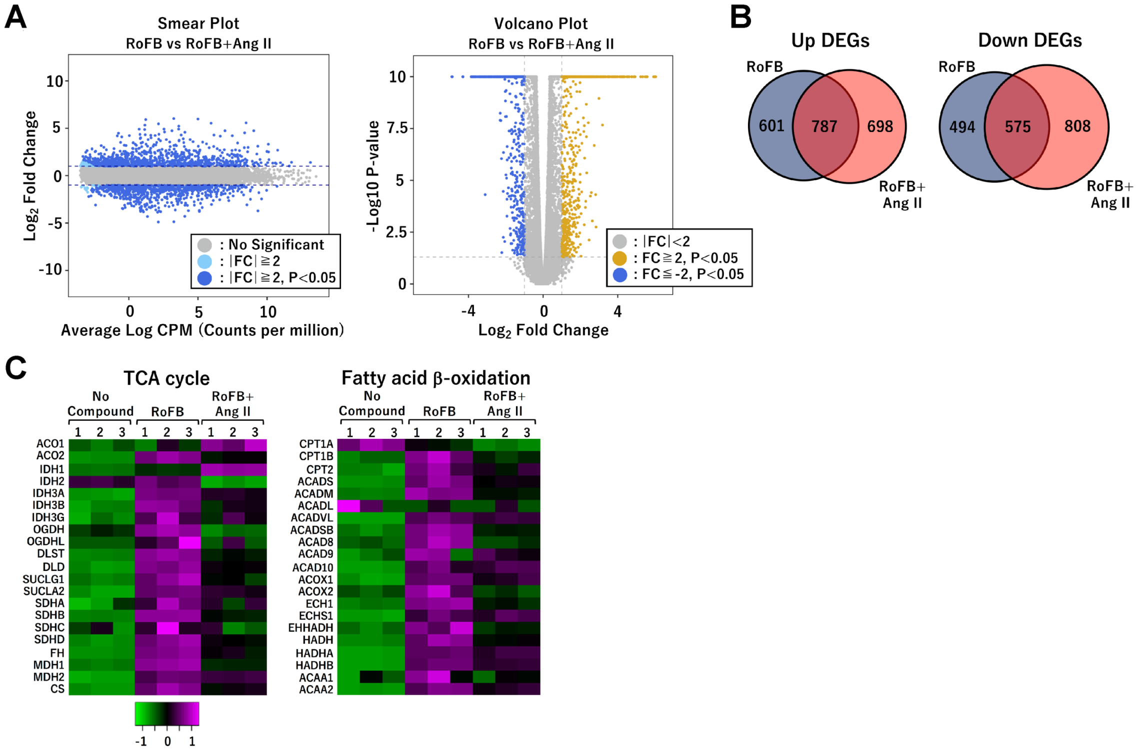

Supplementary Figure S6. Comparison of RNA-Seq analysis between the control ciBAs (RoFB) and ciBAs continuously treated with Ang II (RoFB+Ang II) at 0.1 μM. (**A**) Smear and volcano plots indicate the relation of logarithmic FC, P-value, and CPM (counts per million) between them. (**B**) Venn diagrams represent an overlap of up- and down-regulated DEGs in the control ciBAs and Ang II-treated ciBAs. (**C**) Heat maps represent transcriptional profiles in the functional group of TCA cycle and fatty acid β-oxidation. The color scale shows z-scored fragments per kilobase of transcript per million mapped sequence reads (FPKM) representing mRNA levels of each gene in green (lower expression) and magenta (higher expression).

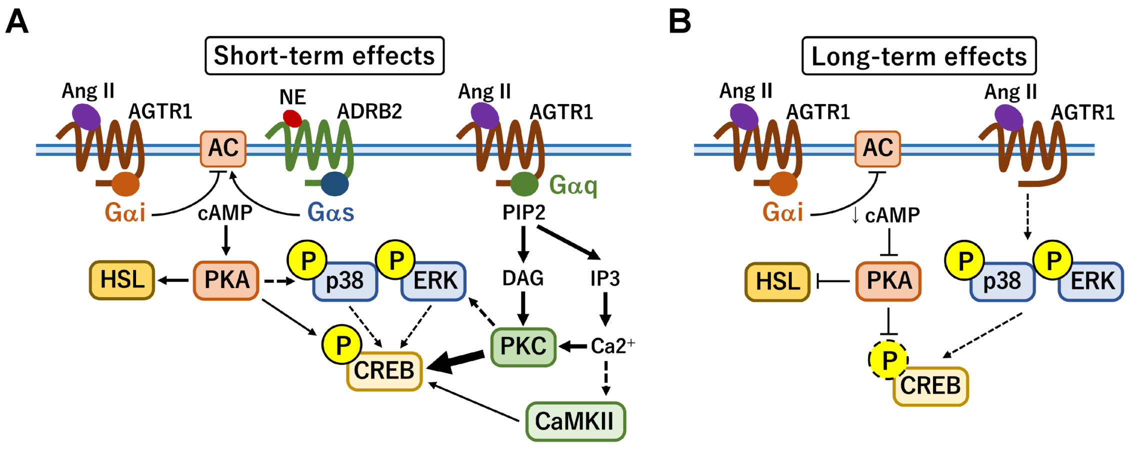

Supplementary Figure S7. A schematic illustration of the regulation of CREB phosphorylation through the Ang II/AGTR1 axis. (**A**) The regulation of CREB phosphorylation by the short-term effects of Ang II. AGTR1 receptor couples to Gαi and Gαq proteins to deactivate adenylyl cyclase (AC) and activate phospholipase C (PLC), respectively. The inhibition of AC activity leads to the reduction of cellular cAMP level and may interfere with the UCP1-dependent thermogenic pathway activated by the β-adrenergic receptor, ADBR2, and an endogenous agonist, NE, in response to cold exposure. The thermogenic pathway activates the phosphorylation of p38 and CREB through the protein kinase A (PKA). In contrast, Ang II treatment phosphorylated CREB by the protein kinase C (PKC) activated by Gαq via the increase of Ca^{2+} and diacylglycerol (DAG). Several studies have shown that the calmodulin-dependent protein kinase II (CaMKII) is involved in Ang II-mediated CREB phosphorylation. Furthermore, CREB was partially phosphorylated by p38 and ERK signaling pathways. PKC may indirectly activate ERK phosphorylation. (**B**) The regulation of CREB phosphorylation by the long-term effects of Ang II. Ang II can reduce cAMP production via the AC-inhibitory Gαi protein coupled to AGTR1. The continuous inhibition of the cAMP/PKA pathway results in the reduced phosphorylation of both HSL and CREB proteins. In contrast, the p38/ERK MAPK pathways are continuously activated by Ang II. Thus, Ang II regulates multiple signaling pathways, which partially overlap with the thermogenic pathway in the human brown adipocyte model.

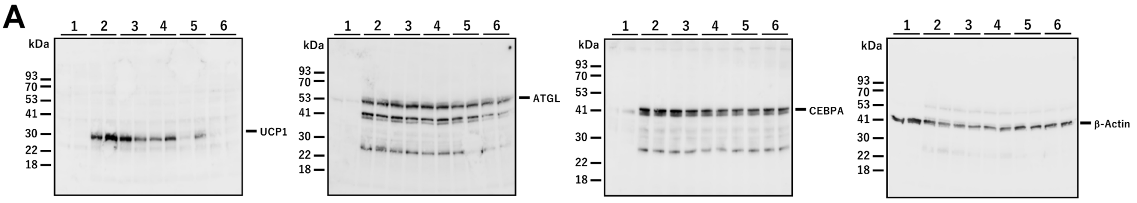

Figure 2A
1: No compound
2: RoFB
3: RoFB+Ang II (0.001 μM)
4: RoFB+Ang II (0.01 μM)
5: RoFB+Ang II (0.1 μM)
6: RoFB+Ang II (1 μM)

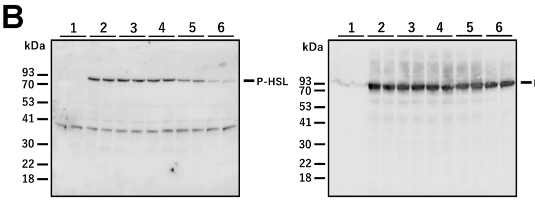

Figure 2G
1: No compound
2: RoFB
3: RoFB+Ang II (0.001 μM)
4: RoFB+Ang II (0.01 μM)
5: RoFB+Ang II (0.1 μM)
6: RoFB+Ang II (1 μM)

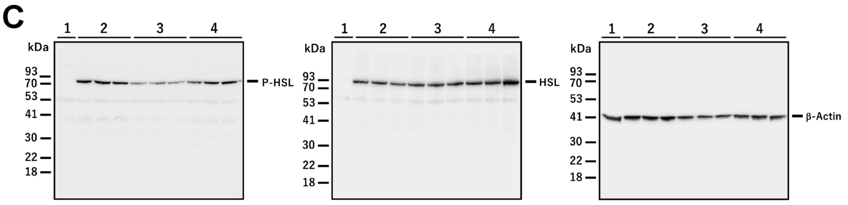

Figure 2H
1: No compound
2: RoFB
3: RoFB+Ang II
4: RoFB+Ang II+telmisartan

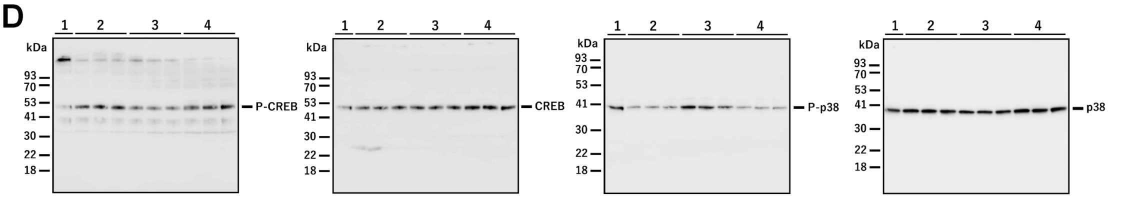

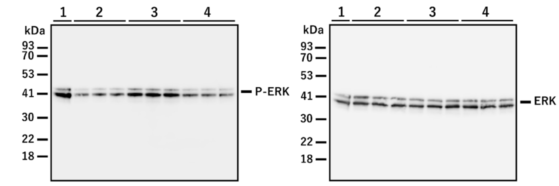

Figure 2I
1: No compound
2: RoFB
3: RoFB+Ang II
4: RoFB+Ang II+telmisartan

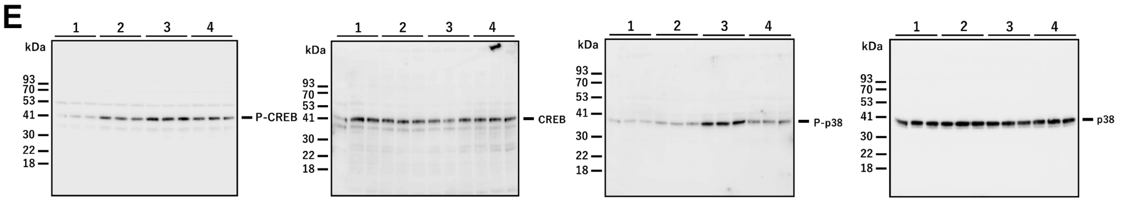

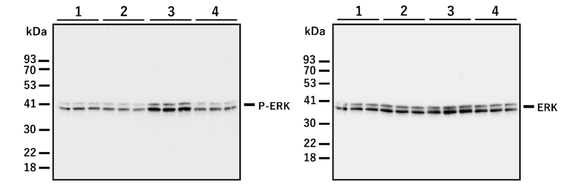

Figure 3E
1: DMSO (30 min)
2: Iso (30 min)
3: Iso+Ang II (30 min)
4: Iso+Ang II+telmisartan (30 min)

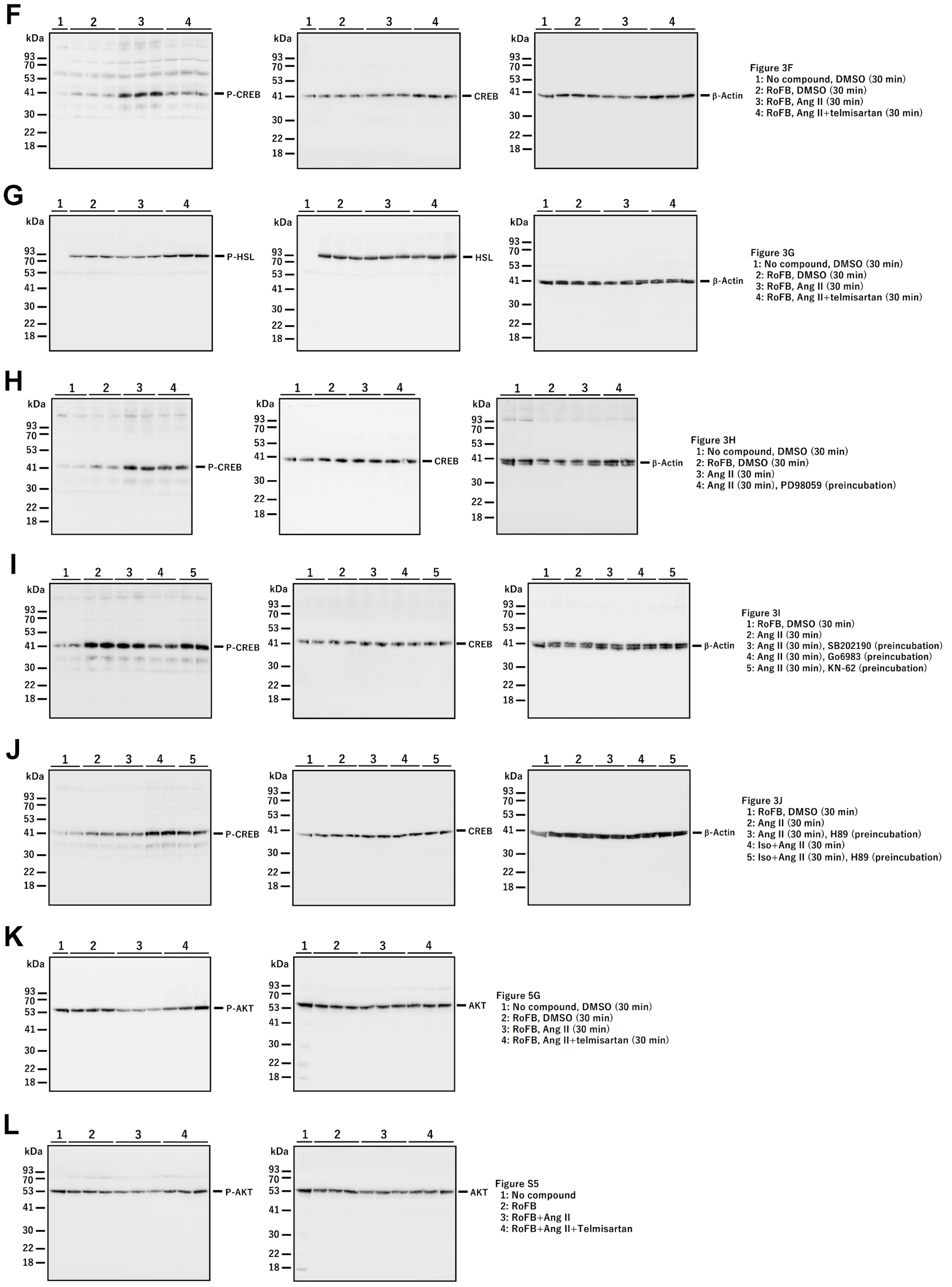

Supplementary Figure S8. (**A-K**) Original images of full-length western blot are shown for Figure 2A (**A**), Figure 2G (**B**), Figure 2H (**C**), Figure 2I (**D**), Figure 3E (**E**), Figure 3F (**F**), Figure 3G (**G**), Figure 3H (**H**), Figure 3I (**I**), Figure 3J (**J**), Figure 5G (**K**), and Figure S5 (**L**).

Supplementary Table 1. List of muscle-related genes downregulated by Ang II treatment in ciBAs.

Gene name	Description	Fold Change	p-value
TRPC3	Transient receptor potential cation channel subfamily C member 3	92.44	9.88.E-04
KCNH2	Potassium voltage-gated channel subfamily H member 2	65.52	2.58.E-04
ADRA1A	Adrenoceptor alpha 1A	60.46	9.64.E-05
STAC2	SH3 and cysteine rich domain 2	60.11	1.84.E-04
MYH11	Myosin heavy chain 11	46.25	3.17.E-04
PLN	Phospholamban	45.59	3.13.E-04
MYH2	Myosin heavy chain 2	35.35	6.36.E-05
DES	Desmin	19.32	9.13.E-04
ASB2	Ankyrin repeat and SOCS box containing 2	18.82	2.72.E-04
CNN1	Calponin 1	17.73	5.14.E-05
IGF1	Insulin like growth factor 1	17.16	2.15.E-05
ITGA8	Integrin subunit alpha 8	10.74	1.37.E-04
ACTA2	Actin alpha 2, smooth muscle	10.35	1.86.E-05
MYH1	Myosin heavy chain 1	9.70	2.26.E-03
NOS1AP	Nitric oxide synthase 1 adaptor protein	9.23	3.51.E-03
TPM1	Tropomyosin 1	7.80	1.02.E-04
LMOD1	Leiomodin 1	7.76	7.29.E-05
KCNJ3	Potassium inwardly rectifying channel subfamily J member 3	7.22	3.35.E-03
MYLK	Myosin light chain kinase	7.04	9.93.E-05
MYOCD	Myocardin	6.47	1.55.E-04
ADRA2C	Adrenoceptor alpha 2C	6.45	7.95.E-05
NPNT	Nephronectin	6.24	7.71.E-03
JUP	Junction plakoglobin	5.98	3.23.E-05
TAGLN	Transgelin	5.59	2.02.E-05
EDNRA	Endothelin receptor type A	5.26	6.93.E-05
TMOD1	Tropomodulin 1	4.97	5.32.E-04
PPP1R12B	Protein phosphatase 1 regulatory subunit 12B	4.48	1.33.E-03
SYNM	Synemin	4.28	4.21.E-04
PROX1	Prospero homeobox 1	4.28	8.60.E-03
PDLIM5	PDZ and LIM domain 5	4.10	1.55.E-04
TMEM38A	Transmembrane protein 38A	3.84	1.32.E-05
FBXO32	F-box protein 32	3.67	4.91.E-05
MYL3	Myosin light chain 3	3.63	1.68.E-02
DTNA	Dystrobrevin alpha	3.62	5.13.E-05
TPM2	Tropomyosin 2	3.18	1.70.E-05
MYL9	Myosin light chain 9	3.15	8.68.E-06
TGFB2	Transforming growth factor beta 2	3.06	5.18.E-04
CASQ2	Calsequestrin 2	3.06	4.98.E-04
SLMAP	Sarcolemma associated protein	2.90	1.84.E-05
SNTA1	Syntrophin alpha 1	2.85	5.20.E-05
SLC8A1	Solute carrier family 8 member A1	2.81	3.01.E-04

HAND2	Heart and neural crest derivatives expressed 2	2.79	1.04.E-04
BMP4	Bone morphogenetic protein 4	2.69	8.10.E-03
ATP1A2	ATPase Na+/K+ transporting subunit alpha 2	2.66	2.67.E-04
SMTN	Smoothelin	2.63	3.46.E-05
KCNJ8	Potassium inwardly rectifying channel subfamily J member 8	2.63	5.70.E-04
FLNA	Filamin A	2.61	2.63.E-04
CACNB2	Calcium voltage-gated channel auxiliary subunit beta 2	2.58	2.42.E-04
IGFBP5	Insulin like growth factor binding protein 5	2.57	3.03.E-05
PKP2	Plakophilin 2	2.53	1.18.E-04
CALD1	Caldesmon 1	2.37	3.10.E-04
CD38	CD38 molecule	2.35	2.98.E-02
P2RY1	Purinergic receptor P2Y1	2.31	1.26.E-03
MYL6	Myosin light chain 6	2.21	1.52.E-05
ACTB	Actin beta	2.15	2.08.E-05
SGCA	Sarcoglycan alpha	2.13	1.22.E-02
ABAT	4-aminobutyrate aminotransferase	2.10	2.97.E-03
TGFBR3	Transforming growth factor beta receptor 3	2.06	6.12.E-06
FOXO1	Forkhead box O1	2.04	2.60.E-04
ACTN1	Actinin alpha 1	2.03	8.17.E-06

Supplementary Table S2. Sequences of primers used for qRT-PCR.

Gene	Sense primer	Antisense primer
TBP	ACTACGGGGTTATCACCTGTGAG	GTGCAGGAGTAGGCCACATTAC
UCP1	TCTACGACACGGTCCAGGAG	GAATACTGCCACTCCTCCAGTC
FABP4	GCCAGGAATTTGACGAAGTCA	CCCATTTCTGCACATGTACCAG
CIDEA	AAGGCCACCATGTATGAGATGTAC	ACAGGAACCGCAGCAGACTC
AGT	CTCCCTCAACTGGATGAAGAAAC	CATAAGATCCTTGCAGCACCAG
HK1	ACACTACGACAGACTGGTGGAC	AGATGTTGCGGACGATTTCAC
HK2	ACAGTCGGAACTATGATGACCTG	CCTCCATGTAGCAGGCATTG
PFKFB3	GCCTGCTTGCCTACTTCCTG	CGACAGGCGTCAGTTTCAG
LDHB	TGGAAGGAAGTGCATAAGATGG	AGATCAGCCACACTTAATCCAATAG
G6PD	GACCTGACCTACGGCAACAG	GCAGAAGACGTCCAGGATGAG
SLC2A1	TCATTGTGGGCATGTGCTTC	ATGAAGAACAGAACCAGGAGCAC
SLC2A4	TGATGACTGTGGCTCTGCTC	CAAAGATGGCCACAATGGAG
PDHA1	TCTCAAGGACAGGATGGTGAAC	CAATCTCCTTCCTCACTTCCAC
PC	GCAAGCACTACTTCATCGAGGTC	TCAGCCACGTGGATCTGAG
CS	ACATCTGGAACACACTCAACTCAG	ACAGGTATATCGCGGATCAGTC
